# Foliar Sodium Selenite Partially Alleviates Drought Injury and Improves Functional Quality in Mulberry Leaves

**DOI:** 10.3390/biology15161368

**Published:** 2026-08-11

**Authors:** Baolong Du, Weigang Fu, Yuan Wang, Juexian Dong, Jinlong Li, Nan Xu, Dawei Guan, Haixiu Zhong

**Affiliations:** 1 Key Laboratory of Heilongjiang Province for Cold-Regions Wetlands Ecology and Environment Research, Harbin University, Harbin 150086, China; 2 Heilongjiang Provincial Forestry and Grassland Survey Planning and Design Institute, Harbin 150086, China; 3 Institute of Natural Resources and Ecology, Heilongjiang Academy of Sciences, Harbin 150040, China

**Keywords:** mulberry leaves, sodium selenite, drought stress, selenium biofortification, photosynthetic performance, functional compounds

## Abstract

Mulberry leaves are used as functional food materials, but drought can reduce leaf production and physiological stability. This study evaluated whether foliar sodium selenite application could partially alleviate drought injury while increasing total selenium and selected functional-quality indicators in mulberry *(Morus alba* L.) leaves. Seedlings were assigned to four treatments: normal water supply with the surfactant-containing control spray (CK), normal water supply with sodium selenite application (Se), drought stress with the control spray (D+CK), and drought stress with sodium selenite application (D+Se). Drought reduced plant growth, leaf water status, leaf pigment status, gas exchange, and PSII photochemical performance while increasing oxidative damage and membrane injury. Under drought stress, sodium selenite application partially maintained growth, water status, photosynthetic performance, and antioxidant enzyme activities and was associated with lower oxidative-damage indicators. Drought alone increased several stress-responsive secondary metabolites but reduced biomass, polysaccharides, and soluble protein, indicating a trade-off rather than a uniform improvement in leaf quality. Compared with D+CK, D+Se increased total selenium, several functional compounds, and in vitro antioxidant capacity. Overall, foliar application of 10 μM sodium selenite was associated with partial drought-stress alleviation and higher functional-quality indicators under controlled pot conditions. Further studies are required to optimize the application dose and evaluate selenium speciation, bioaccessibility, intake safety, and field performance.

## Simple Summary

Drought can reduce mulberry leaf production and quality by limiting water supply, photosynthesis, and normal cell function. Selenium is needed in small amounts in the human diet and may also help plants tolerate environmental stress. This study tested whether spraying mulberry leaves with a low concentration of sodium selenite could reduce drought injury and improve the food-related quality of the leaves. Under controlled pot conditions, drought reduced plant growth, leaf water content, photosynthetic performance, and cell membrane stability. Plants that received selenium during drought maintained better growth and photosynthesis, accumulated fewer damaging oxygen-related molecules, and showed stronger natural protective responses than drought-stressed plants without selenium. Foliar sodium selenite application also increased leaf selenium content and several compounds associated with antioxidant capacity and potential food-related value. These findings suggest that low-dose foliar sodium selenite application may partially protect mulberry seedlings from drought while improving selected leaf-quality traits. Further studies are needed to identify the best dose, determine which forms of selenium are present, assess how much can be absorbed by the human body, confirm product safety, and test the treatment under field conditions before practical use.

## 1. Introduction

Mulberry leaves (*Morus alba* L.) have gained attention as functional food ingredients because they contain phenolics, flavonoids, polysaccharides, alkaloids, and phenolic acids. Of these compounds, 1-deoxynojirimycin (1-DNJ) is a typical iminosugar of mulberry leaves and has drawn interest due to its importance in carbohydrate metabolism and functional food formulation [1,2,3]. In addition, mulberry leaves also include chlorogenic acid, rutin, quercetin and other phenolic compounds to enhance the antioxidant activity and nutritional values [4,5,6,7]. Thus, there is a need to enhance the stress tolerance as well as accumulation of functional compounds of mulberry leaves to create high-quality plant-based food materials.

Water shortage is an important abiotic factor that influences plant growth, photosynthesis, and metabolite accumulation. Drought may decrease leaf development, biomass accumulation, water status, and leaf yield in mulberry, thereby constraining the production of functional leaf materials [8]. In mulberry, water deficit restricts leaf expansion and biomass accumulation and may visibly cause reduced leaf size and greenness, loss of turgor, leaf or petiole drooping, wilting, and premature senescence [8,9,10]. These visible responses are commonly accompanied by lower leaf water and chlorophyll contents, restricted stomatal conductance and CO_2_ assimilation, impaired photosynthetic electron transport, and greater oxidative and membrane injury. Drought may nevertheless stimulate the accumulation of some stress-responsive secondary metabolites, particularly phenolics and flavonoids. Mulberry cultivation remains an important component of China’s sericulture sector. Official national statistics reported 11.895 million mu, approximately 793,000 ha, of mulberry plantations and 620,000 t of silkworm cocoon production in 2016 [11]. Because leaf yield and quality directly affect sericultural productivity and the supply of leaves for food-oriented uses, drought during shoot and leaf development can adversely affect mulberry production. Therefore, the effect of drought on mulberry-leaf quality represents a trade-off between reduced growth and physiological stability and increased accumulation of selected secondary metabolites.

Photosynthesis supports biomass production and supplies carbon for metabolite biosynthesis. Drought can restrict CO_2_ assimilation through both stomatal and non-stomatal limitations. Gas-exchange and chlorophyll-fluorescence measurements were, therefore, used to evaluate carbon assimilation and photosystem II (PSII) photochemical performance under drought stress.

Selenium is an essential dietary trace element and can act as a beneficial element in plants when supplied at low concentrations. Its effects on plant drought responses are nevertheless dependent on crop species, selenium form, application dose, and stress intensity [12,13]. In tomato, foliar application of 2.5 mg L^−1^ sodium selenite reduced reactive oxygen species (ROS) and malondialdehyde accumulation and helped maintain photosynthesis and dry-matter accumulation under drought, whereas higher concentrations produced less favorable responses [14]. In camelina and canola, Ahmad et al. (2021) evaluated 7.06 μM foliar sodium selenite alone and in combination with 75 μM sodium selenite seed priming; the combined treatment improved water relations, antioxidant responses, and yield-related traits under water deficit [15]. These findings support the evaluation of low-dose sodium selenite as a potential drought-mitigation treatment but also indicate that its effects cannot be generalized across concentrations and crop species.

Foliar application can also contribute to agronomic selenium biofortification because selenium absorbed by leaves can be redistributed to harvested edible organs. In strawberry, foliar sodium selenite at 10–100 mg L^−1^ increased selenium accumulation in fruits, and the 40 mg L^−1^ treatment improved several fruit-quality traits [16]. In wheat, foliar selenite application at 15 g Se ha^−1^ increased grain selenium concentration, and organic selenium was the predominant form detected in the grain [17]. Selenium is incorporated into human selenoproteins involved in thyroid hormone metabolism and protection against oxidative damage. The recommended dietary allowance for selenium is 55 μg d^−1^ for most adults [18]. A human dietary intervention study showed that consumption of naturally selenium-rich Brazil nuts increased plasma selenium concentration and glutathione peroxidase activity, supporting the potential of selenium-rich foods to improve selenium status [19]. However, this dietary reference value should not be interpreted as a target selenium concentration for an individual food product. The nutritional value and intake safety of selenium-enriched mulberry leaves should be evaluated together with selenium speciation, bioaccessibility, serving size, processing stability, background dietary intake, and total selenium exposure.

Regardless of growing awareness of the use of mulberry leaves as functional food ingredients and the application of selenium biofortification as a nutritional approach, there is scant data on the effect of exogenous selenium on drought tolerance and functional characteristics of mulberry leaves. The majority of earlier works have been devoted to mulberry phytochemistry and antioxidant activity under typical conditions or to plant stress physiology with no connection between stress reduction and food-quality characteristics [20,21,22]. The question of whether the use of selenium may at the same time reduce the physiological damage caused by drought, increase selenium content, preserve the photosynthesis capacity, and enhance the synthesis of active substances, including phenolics, flavonoids, 1-DNJ, polysaccharides, chlorogenic acid, rutin and quercetin is still unanswered.

Accordingly, this study had two objectives: (1) to evaluate whether foliar sodium selenite application partially alleviates drought-related physiological and oxidative injury in ‘Longsang No. 1’ mulberry seedlings and (2) to determine whether this treatment increases total selenium and selected functional-quality indicators in mulberry leaves. We hypothesized that foliar sodium selenite application would partially maintain physiological performance under drought stress while increasing total selenium and selected functional-quality indicators.

## 2. Materials and Methods

### 2.1. Plant Material and Growth Conditions

Uniform nursery-grown seedlings of mulberry (*Morus alba* L. ‘Longsang No. 1’) were used in this study. The experiment was conducted in 2025 as a single 30 d controlled pot experiment. ‘Longsang No. 1’ was selected because it is a regionally relevant mulberry cultivar used for leaf and fruit production in northern China and because uniform nursery-grown seedlings were locally available in Heilongjiang Province. A previous comparison of four mulberry clones reported relatively favorable drought performance of ‘Longsang No. 1’ under the specific experimental conditions, although this does not support its general classification as either drought-tolerant or drought-susceptible [23]. Only one cultivar was included to provide a consistent genetic background for evaluating the effects of water regime and foliar sodium selenite application. The cultivar was not selected on the basis of established superiority in selenium accumulation, and the experiment was not designed to compare genotype-specific drought responses or selenium-enrichment capacity. Before treatment, seedlings with similar growth status were selected. The initial plant height was approximately 50–55 cm, and the basal stem diameter was approximately 5.0 ± 0.5 mm. Seedlings showing visible disease symptoms, mechanical damage, or abnormal growth were excluded.

Each seedling was transplanted into a 3.0 L plastic pot with drainage holes. Each pot was filled with a sterilized substrate mixture of peat, perlite, and vermiculite at a volume ratio of 3:1:1. Before treatment, all seedlings were acclimated for 10 d under controlled environmental conditions with a 14 h/10 h light/dark photoperiod, day/night temperatures of 25/18 °C, relative humidity of 65–75%, and a photosynthetic photon flux density of approximately 250 μmol m^−2^ s^−1^. During acclimation, plants were watered regularly to avoid water deficit. Drought and sodium selenite treatments were initiated immediately after the 10 d acclimation period.

Fully expanded mature leaves at the same developmental position were collected after treatment for physiological, biochemical, selenium-enrichment, phytochemical, and antioxidant-capacity assays.

### 2.2. Experimental Design and Treatments

The experiment was arranged as a two-factor factorial design with water regime and sodium selenite application as the two factors. Four treatment combinations were established: normal water supply with the surfactant-containing control spray (CK), normal water supply with foliar sodium selenite application (Se), drought stress with the surfactant-containing control spray (D+CK), and drought stress with foliar sodium selenite application (D+Se). Following the 10 d acclimation period, normal-water treatments were maintained at 70–75% of substrate field capacity, whereas drought treatments were maintained at 40–45% of substrate field capacity throughout the 30 d treatment period.

Substrate field capacity was determined before treatment using representative pots filled with the same substrate mixture. The substrate was saturated with water, and the pots were allowed to drain freely for 24 h. The pot weight recorded after drainage was defined as the field-capacity weight. During the 30 d treatment period, each pot was weighed once daily at the same time. Distilled water was added as required to restore normal-water pots to 70–75% and drought-treated pots to 40–45% of substrate field capacity.

Sodium selenite (Na_2_SeO_3_, analytical grade) was applied foliarly at 10 μM, corresponding to approximately 0.79 mg Se L^−1^. The experiment was designed as a single-concentration factorial evaluation rather than as a dose–response study. The 10 μM treatment was selected as a conservative low-dose treatment because selenium responses are concentration dependent and excessive application may adversely affect plant performance. Therefore, the concentration used in this study should not be interpreted as an optimal application dose. The sodium selenite solution contained 0.05% Tween-20 as a surfactant. Plants assigned to CK and D+CK received a control solution consisting of distilled water with the same concentration of Tween-20. The sodium selenite or control solution was sprayed evenly onto both sides of the leaves until runoff. The first application was performed 24 h before drought initiation, and the second application was performed on treatment day 7. The treatment period lasted 30 d.

Each treatment contained six biological replicates, with one potted plant considered one biological replicate. Pots were randomly arranged and repositioned every two days to reduce positional effects. At the end of the 30 d treatment, SPAD, gas-exchange, and chlorophyll-fluorescence measurements were completed on fully expanded mature leaves at the same developmental position. Leaves were then harvested separately from each plant, with each plant retained as one biological replicate. Fresh subsamples were used immediately for leaf relative water content, photosynthetic pigment, soluble sugar, and soluble protein determinations. Subsamples used for oxidative-damage and antioxidant-enzyme assays were immediately frozen in liquid nitrogen and stored at −80 °C until analysis. For total selenium determination, leaf material was dried and ground into fine powder. For functional-compound and antioxidant-capacity analyses, leaf samples were immediately frozen in liquid nitrogen, freeze-dried, ground to pass through a 60-mesh sieve, and stored at −80 °C until extraction.

### 2.3. Growth Traits and Leaf Water Status

After the 30 d treatment period, plant height, mean single-leaf area, shoot fresh weight, shoot dry weight, and leaf relative water content were measured. Plant height was measured from the substrate surface to the shoot apex using a ruler. Mean leaf area was measured using an LI-3000C portable leaf area meter (LI-COR Biosciences, Lincoln, NE, USA). Shoots were harvested and weighed immediately to obtain shoot fresh weight. Samples were then heated at 105 °C for 30 min and dried at 75 °C to constant weight to determine shoot dry weight.

Leaf relative water content was determined using the relative turgidity method described by Barrs and Weatherley (1962) [24]. Fresh leaf discs were weighed immediately after sampling to obtain the fresh weight (FW). The discs were floated in distilled water in darkness for 6 h and then gently blotted before measurement of the turgid weight (TW). They were subsequently dried at 75 °C to constant weight to obtain the dry weight (DW). Leaf relative water content was calculated as follows:

RWC (%) = [(FW − DW)/(TW − DW)] × 100

### 2.4. SPAD Value and Photosynthetic Pigments

SPAD values were measured using a SPAD-502 Plus chlorophyll meter (Konica Minolta, Tokyo, Japan). Five readings were taken from fully expanded mature leaves of each biological replicate, avoiding the main vein, and the mean value was used for analysis.

Fresh leaf tissue (0.20 g) was extracted with 20 mL of 80% acetone in darkness until the tissue was completely decolorized. The extract was centrifuged at 10,000× *g* for 10 min, and absorbance was measured at 663, 646, and 470 nm using a UV-1800 UV–visible spectrophotometer (Shimadzu, Kyoto, Japan). The concentrations of chlorophyll a (C_a_), chlorophyll b (C_β_), and total carotenoids (C_x+c_) in the extract were calculated according to Lichtenthaler and Wellburn (1983) using the following Equations [25]:

C_a_ = 12.21A_663_ − 2.81A_646_

C_β_ = 20.13A_646_ − 5.03A_663_

C_x+c_ = (1000A_470_ − 3.27C_a_ − 104C_β_)/229

where A_663_, A_646_, and A_470_ represent the absorbance values measured at 663, 646, and 470 nm, respectively. C_a_, C_β_, and C_x+c_ are the concentrations of chlorophyll a, chlorophyll b, and total carotenoids in the extract, respectively, expressed as μg mL^−1^. The content of each pigment on a fresh-weight basis was calculated as follows:

Pigment content (mg g^−1^ FW) = C × V/(1000 × m)

where C is the pigment concentration in the extract (μg mL^−1^), V is the total extract volume (mL), and m is the fresh mass of the leaf sample (g). Reagent blanks were used for absorbance correction.

### 2.5. Gas-Exchange Measurements

Leaf gas-exchange parameters were measured in June 2025, on day 30 of the treatment, between 09:00 and 11:30 h local time in the morning using a CIRAS-3 portable photosynthesis system (PP Systems, Amesbury, MA, USA). Fully expanded mature leaves at the same developmental position were selected for measurement. The chamber conditions were set as follows: photosynthetic photon flux density, 1000 μmol m^−2^ s^−1^; CO_2_ concentration, 400 μmol mol^−1^; leaf temperature, 25 °C and relative humidity, 60–70%.

Readings were recorded after the net photosynthetic rate had remained stable for at least 2 min. The measured parameters included net photosynthetic rate (Pn, μmol CO_2_ m^−2^ s^−1^), stomatal conductance (g_s_, mol H_2_O m^−2^ s^−1^), intercellular CO_2_ concentration (Ci, μmol CO_2_ mol^−1^), and transpiration rate (Tr, mmol H_2_O m^−2^ s^−1^). Instantaneous water-use efficiency was calculated as follows:

WUE = Pn/Tr

where WUE was expressed as μmol CO_2_ mmol^−1^ H_2_O. Gas-exchange interpretation followed standard principles for distinguishing stomatal and non-stomatal photosynthetic limitations [26,27].

### 2.6. Chlorophyll Fluorescence Measurements

Chlorophyll fluorescence parameters were measured using an FMS-2 pulse-modulated chlorophyll fluorometer (Hansatech Instruments Ltd., King’s Lynn, UK). Leaves were dark-adapted for 30 min before measurement. Minimum fluorescence (Fo) was recorded under weak measuring light, and maximum fluorescence (Fm) was obtained using a saturating pulse. The maximum quantum efficiency of PSII was calculated as follows:

Fv/Fm = (Fm − Fo)/Fm

After dark-adapted measurements, leaves were exposed to actinic light at 600 μmol m^−2^ s^−1^ for 5 min. Steady-state fluorescence (Fs) and maximum fluorescence under light (Fm′) were recorded. The effective quantum yield of PSII Y(II), ETR, NPQ, and qP were obtained directly from the instrument software (FMS Systems Software V3.11) based on the recorded fluorescence signals and the actual measurement settings.

### 2.7. Oxidative-Damage Indicators, Relative Electrolyte Leakage, and Antioxidant Enzyme Activities

Frozen leaf tissue (0.50 g) was homogenized with 5 mL of ice-cold 50 mM potassium phosphate buffer (pH 7.0) containing 1 mM EDTA and 1% polyvinylpolypyrrolidone. The homogenate was centrifuged at 12,000× *g* for 15 min at 4 °C, and the supernatant was used for antioxidant enzyme assays.

Hydrogen peroxide (H_2_O_2_) content was determined using the titanium sulfate method and expressed as μmol g^−1^ FW [28,29]. The superoxide anion (O_2_^−^) production rate was measured using the hydroxylamine method and expressed as nmol g^−1^ FW min^−1^ [30]. Malondialdehyde (MDA) content was determined using the thiobarbituric acid method and expressed as nmol g^−1^ FW [31].

Relative electrolyte leakage (REL) was determined using a DDS-307A conductivity meter (Leici, Shanghai, China). Leaf discs were immersed in deionized water for 12 h, and the initial conductivity was recorded as C1. The samples were then boiled for 20 min, cooled to room temperature, and the final conductivity was recorded as C2. REL was calculated as follows:

REL (%) = C1/C2 × 100

Superoxide dismutase (SOD) activity was determined from the inhibition of nitroblue tetrazolium photoreduction at 560 nm [32]. Peroxidase (POD) activity was measured using the guaiacol–H_2_O_2_ method by monitoring the increase in absorbance at 470 nm [33]. Catalase (CAT) activity was determined by monitoring H_2_O_2_ decomposition at 240 nm [34]. Ascorbate peroxidase (APX) activity was measured by monitoring the decrease in absorbance at 290 nm caused by ascorbate oxidation [35].

### 2.8. Determination of Soluble Sugar and Soluble Protein

Soluble sugar content was determined using the anthrone–sulfuric acid method. Fresh leaf tissue (0.50 g) was extracted with distilled water in a boiling water bath. The extract was reacted with anthrone reagent and concentrated sulfuric acid, and absorbance was measured at 620 nm using a UV-1800 UV–visible spectrophotometer (Shimadzu, Kyoto, Japan). Soluble sugar content was expressed as mg g^−1^ FW.

Soluble protein content was determined using the Coomassie Brilliant Blue G-250 method [36]. An aliquot of the phosphate-buffer extract prepared from 0.50 g of fresh leaf tissue with a final extract volume of 5 mL was reacted with Coomassie Brilliant Blue G-250 reagent. Absorbance was measured at 595 nm using a UV-1800 UV–visible spectrophotometer (Shimadzu, Kyoto, Japan), and bovine serum albumin was used to prepare the calibration curve. The calibration relationship was expressed as follows:

A595 = aC + b

Protein concentration in the extract was calculated as follows:

C = (A595 − b)/a

Soluble protein content was calculated as follows:

Soluble protein content (mg g^−1^ FW) = C × V × D/m

where A595 is the absorbance at 595 nm; a and b are the slope and intercept of the bovine serum albumin calibration curve, respectively; C is the protein concentration in the extract (mg mL^−1^); V is the total extract volume (mL); D is the dilution factor and m is the fresh mass of the leaf sample (g). The results were expressed as mg g^−1^ FW.

### 2.9. Determination of Total Selenium

For total selenium determination, dried mulberry leaves were ground into fine powder. Approximately 0.20 g of dried sample was digested with 6 mL of HNO_3_ and 2 mL of H_2_O_2_ using a MARS 6 microwave digestion system (CEM Corporation, Matthews, NC, USA). After digestion, the solution was cooled, filtered, and diluted to a defined volume with ultrapure water.

Total selenium concentration was determined using an Agilent 7900 inductively coupled plasma mass spectrometer (ICP-MS; Agilent Technologies, Santa Clara, CA, USA). A multi-point selenium calibration curve covering 0–20 μg L^−1^ was used for quantification. Reagent blanks were included during digestion and analysis. Total selenium content was expressed as mg kg^−1^ DW.

### 2.10. Determination of Functional Compounds in Mulberry Leaves

Fresh mulberry leaves were collected after treatment, immediately frozen in liquid nitrogen, freeze-dried, ground to pass through a 60-mesh sieve, and stored at −80 °C until analysis. Unless otherwise stated, results were expressed on a dry-weight basis.

For total phenolics and total flavonoids, 0.50 g of freeze-dried powder was extracted with 25 mL of 70% ethanol by ultrasonication at 40 °C for 30 min. The extract was centrifuged at 10,000× *g* for 10 min, and the supernatant was collected. Extraction was repeated twice, and the combined supernatants were used for analysis. Total phenolic content was determined using the Folin–Ciocalteu method and expressed as mg gallic acid equivalents g^−1^ DW [37]. Total flavonoid content was determined using the aluminum chloride colorimetric method and expressed as mg rutin equivalents g^−1^ DW [38].

Polysaccharides were extracted from 0.50 g of freeze-dried powder with 25 mL of distilled water at 90 °C for 2 h. The extract was centrifuged at 10,000× *g* for 10 min, concentrated, and precipitated with four volumes of 95% ethanol at 4 °C overnight. The precipitate was collected and dissolved in distilled water. Polysaccharide content was determined using the phenol–sulfuric acid method and expressed as mg glucose equivalents g^−1^ DW [39].

HPLC analysis was performed using a Waters e2695 HPLC system (Waters, Milford, MA, USA) equipped with fluorescence and UV detectors and a C18 column (250 mm × 4.6 mm, 5 μm).

1-Deoxynojirimycin (1-DNJ) was determined after pre-column derivatization with 9-fluorenylmethyl chloroformate (FMOC-Cl), following published methods for mulberry leaves with minor modifications [1,2,40]. Freeze-dried leaf powder (0.10 g) was extracted twice with 10 mL of 0.05 M HCl by ultrasonication for 20 min at room temperature. The extracts were centrifuged at 10,000× *g* for 10 min, combined, and diluted to volume with 0.05 M HCl. For derivatization, 100 μL of the extract or 1-DNJ standard solution was mixed with 100 μL of 0.4 M borate buffer (pH 8.5) and 100 μL of 5 mM FMOC-Cl in acetonitrile. The mixture was reacted at 25 °C for 20 min in darkness, followed by the addition of 700 μL of 0.1% acetic acid. The derivatized solution was filtered through a 0.22 μm membrane before HPLC analysis. The mobile phase consisted of 0.1% acetic acid in water and acetonitrile at 55:45 (*v*/*v*). The flow rate was 1.0 mL min^−1^, the column temperature was 30 °C, and the injection volume was 10 μL. Fluorescence detection was performed at excitation and emission wavelengths of 254 and 322 nm, respectively. The 1-DNJ content was quantified using an external 1-DNJ standard curve and expressed as mg g^−1^ DW.

For chlorogenic acid, rutin, and quercetin analysis, freeze-dried leaf powder (0.20 g) was extracted with 10 mL of 70% methanol containing 0.1% formic acid by ultrasonication at 40 °C for 30 min. The extract was centrifuged at 10,000× *g* for 10 min and filtered through a 0.22 μm membrane before HPLC analysis. The mobile phases consisted of 0.1% formic acid in water and acetonitrile. The flow rate was 1.0 mL min^−1^, the column temperature was 30 °C, and the injection volume was 10 μL. Chlorogenic acid, rutin, and quercetin were detected using the UV detector at 327, 360, and 370 nm, respectively, and quantified using the corresponding external standards. The results were expressed as mg g^−1^ DW.

### 2.11. Antioxidant Capacity Assays

DPPH, ABTS, and FRAP assays were used to assess the antioxidant capacity of mulberry leaf extracts. The same ethanolic extract prepared for phenolic analysis was used for antioxidant assays. Extracts were diluted when necessary to ensure that absorbance values were within the linear range of the standard curves. Reagent blanks and standard calibration curves were included in all assays.

For the DPPH assay, the extract was mixed with DPPH solution, incubated in darkness at room temperature, and absorbance was measured at 517 nm. DPPH antioxidant capacity was expressed as μmol Trolox equivalents g^−1^ DW [41].

For the ABTS assay, ABTS radical cation solution was prepared before measurement, mixed with extract, and absorbance was measured at 734 nm. ABTS antioxidant capacity was expressed as μmol Trolox equivalents g^−1^ DW [42].

For the FRAP assay, the extract was mixed with freshly prepared FRAP working solution, and absorbance was measured at 593 nm. FRAP antioxidant capacity was expressed as μmol Fe^2+^ equivalents g^−1^ DW [43].

DPPH and ABTS assess radical-scavenging capacity in different reaction systems, whereas FRAP measures ferric-ion-reducing power. Therefore, the combined use of these assays provides complementary information on the in vitro antioxidant capacity of mulberry leaf extracts.

### 2.12. Statistical Analysis

All data were expressed as mean ± SD based on six biological replicates. Statistical analyses were performed using SPSS 26.0. Data normality and homogeneity of variance were examined using the Shapiro–Wilk test and Levene’s test, respectively. Percentage variables, including leaf relative water content and relative electrolyte leakage, were transformed when necessary before analysis.

Because the experiment included two factors, water regime and sodium selenite application, two-way ANOVA was first used to evaluate the main effects of drought stress, selenium treatment, and their interaction. When significant effects were detected, one-way ANOVA followed by Tukey’s honestly significant difference test was used to compare the four treatment means at *p* < 0.05.

Figures were prepared using OriginPro 2024. Different lowercase letters in figures indicate significant differences among treatments. Full statistical outputs are provided in the Supplementary Materials. Table S1 reports the two-way ANOVA results for water regime, sodium selenite application, and their interaction. Table S2 reports the one-way ANOVA results for the four treatment combinations and supports the lowercase letters shown in the figures.

## 3. Results

### 3.1. Growth and Leaf Water Status

**Figure 1:**
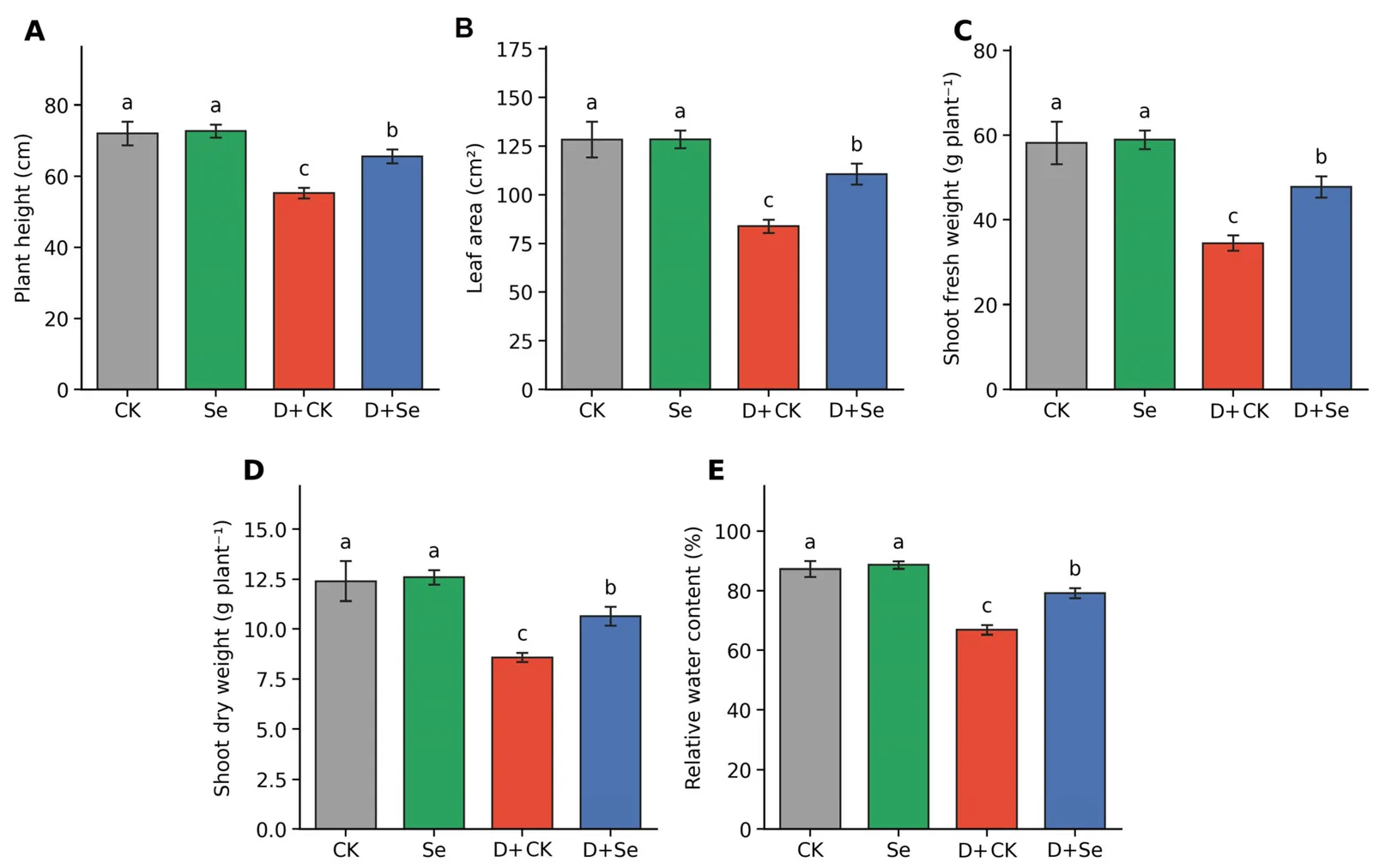
Effects of foliar sodium selenite application on growth and leaf water status of mulberry seedlings under drought stress. Effects of different treatments on (**A**) plant height, (**B**) leaf area, (**C**) shoot fresh weight, (**D**) shoot dry weight, and (**E**) leaf relative water content. CK, normal water supply plus water spray; Se, normal water supply with foliar sodium selenite application; D+CK, drought stress plus water spray; D+Se, drought stress plus selenium spray. Values are means ± SD (n = 6). Different lowercase letters indicate significant differences among treatments according to Tukey’s HSD test at *p* < 0.05.

Foliar sodium selenite application partially alleviated these drought-related decreases. Compared with CK, D+CK decreased plant height, leaf area, shoot fresh weight, shoot dry weight, and leaf relative water content by 23.3%, 34.7%, 40.6%, 30.8%, and 23.4%, respectively (Figure 1A–E). Two-way ANOVA showed significant effects of water regime, sodium selenite application, and their interaction on plant height, leaf area, shoot fresh weight, shoot dry weight, and leaf relative water content (Table S1).

Foliar sodium selenite application partially alleviated the drought-related decreases in growth and leaf water status. Compared with D+CK, D+Se increased plant height, leaf area, shoot fresh weight, shoot dry weight, and leaf relative water content by 18.6%, 32.0%, 38.4%, 24.0%, and 18.5%, respectively. Under normal water supply, most growth variables were comparable between CK and Se, indicating that the response to sodium selenite application was more evident under drought stress.

### 3.2. Leaf Pigment Status and Gas Exchange

**Figure 2:**
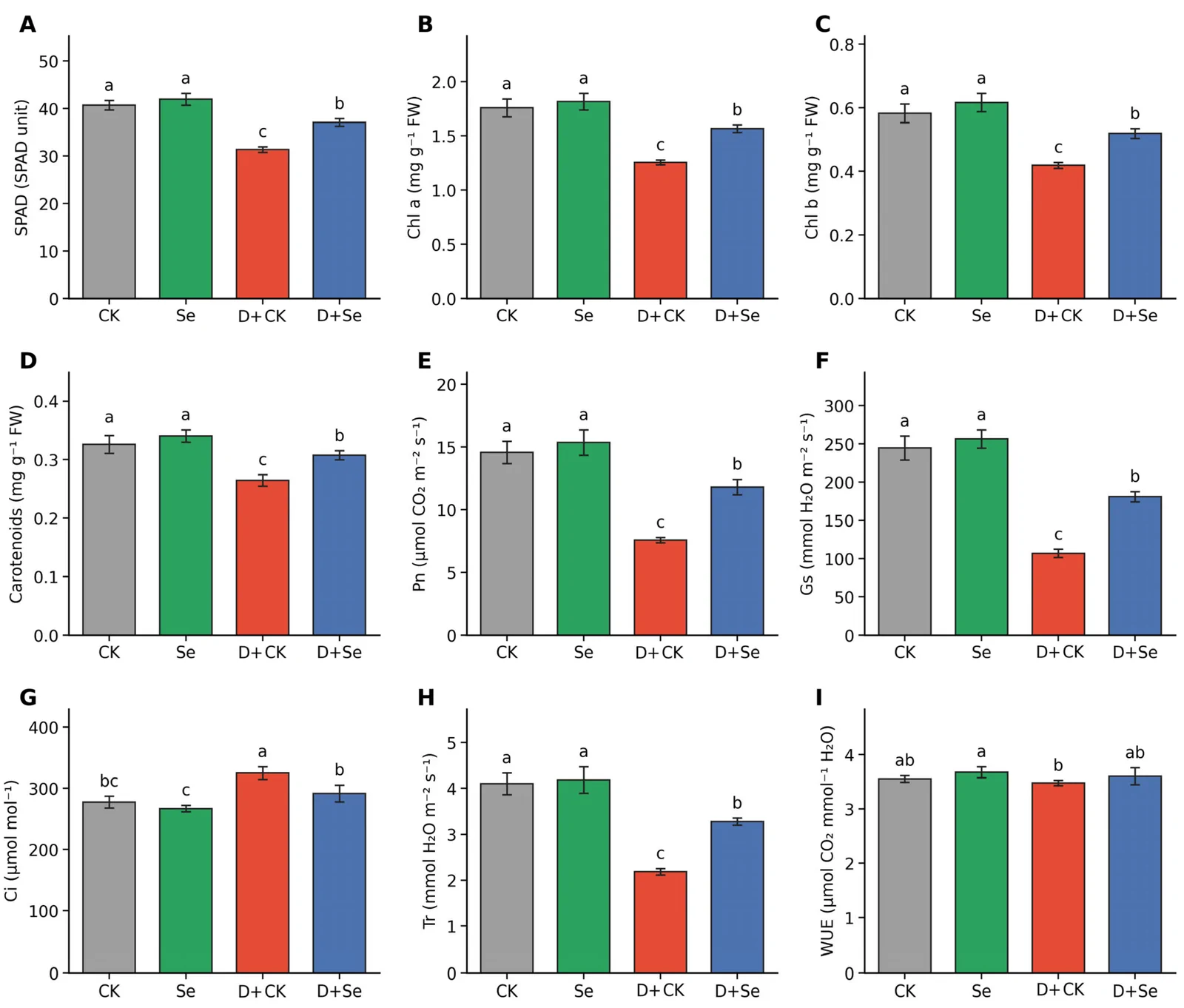
Effects of foliar sodium selenite application on leaf pigment status and gas-exchange parameters of mulberry leaves under drought stress. Effects of different treatments on (**A**) SPAD value, (**B**) chlorophyll a, (**C**) chlorophyll b, (**D**) carotenoids, (**E**) net photosynthetic rate (Pn), (**F**) stomatal conductance (g_s_), (**G**) intercellular CO_2_ concentration (Ci), (**H**) transpiration rate (Tr), and (**I**) instantaneous water-use efficiency (WUE). Values are means ± SD (n = 6). Different lowercase letters indicate significant differences among treatments according to Tukey’s HSD test at *p* < 0.05.

Drought stress significantly reduced the SPAD value and photosynthetic pigment contents in mulberry leaves. Compared with CK, D+CK decreased the SPAD value and the contents of chlorophyll a, chlorophyll b, and carotenoids by 23.0%, 28.6%, 28.1%, and 18.9%, respectively (Figure 2A–D). Foliar sodium selenite application partially maintained leaf pigment status under drought stress. Compared with D+CK, D+Se increased the SPAD value and the contents of chlorophyll a, chlorophyll b, and carotenoids by 18.2%, 24.9%, 24.0%, and 16.3%, respectively. Two-way ANOVA showed significant effects of water regime and sodium selenite application on leaf pigment status (SPAD value, chlorophyll a, chlorophyll b, and carotenoids) and gas-exchange parameters (Pn, g_s_, Ci, Tr, and WUE) (Table S1). The water regime × sodium selenite application interaction was significant for all these variables except WUE.

Gas-exchange parameters showed that drought strongly inhibited photosynthetic carbon assimilation. Compared with CK, D+CK reduced net photosynthetic rate (Pn), stomatal conductance (g_s_), and transpiration rate (Tr) by 48.0%, 56.3%, and 46.8%, respectively (Figure 2E,F,H). In contrast, intercellular CO_2_ concentration (Ci) increased by 17.2% under D+CK (Figure 2G), suggesting that drought-induced photosynthetic inhibition involved non-stomatal limitations in addition to stomatal restriction. WUE changed only slightly among treatments, although Se treatment showed the highest value under normal water supply (Figure 2I).

Foliar sodium selenite application partially maintained gas-exchange performance under drought stress. Compared with D+CK, D+Se increased Pn, g_s_, and Tr by 55.7%, 69.3%, and 50.2%, respectively, while decreasing Ci by 10.3%.

### 3.3. PSII Photochemical Performance

**Figure 3:**
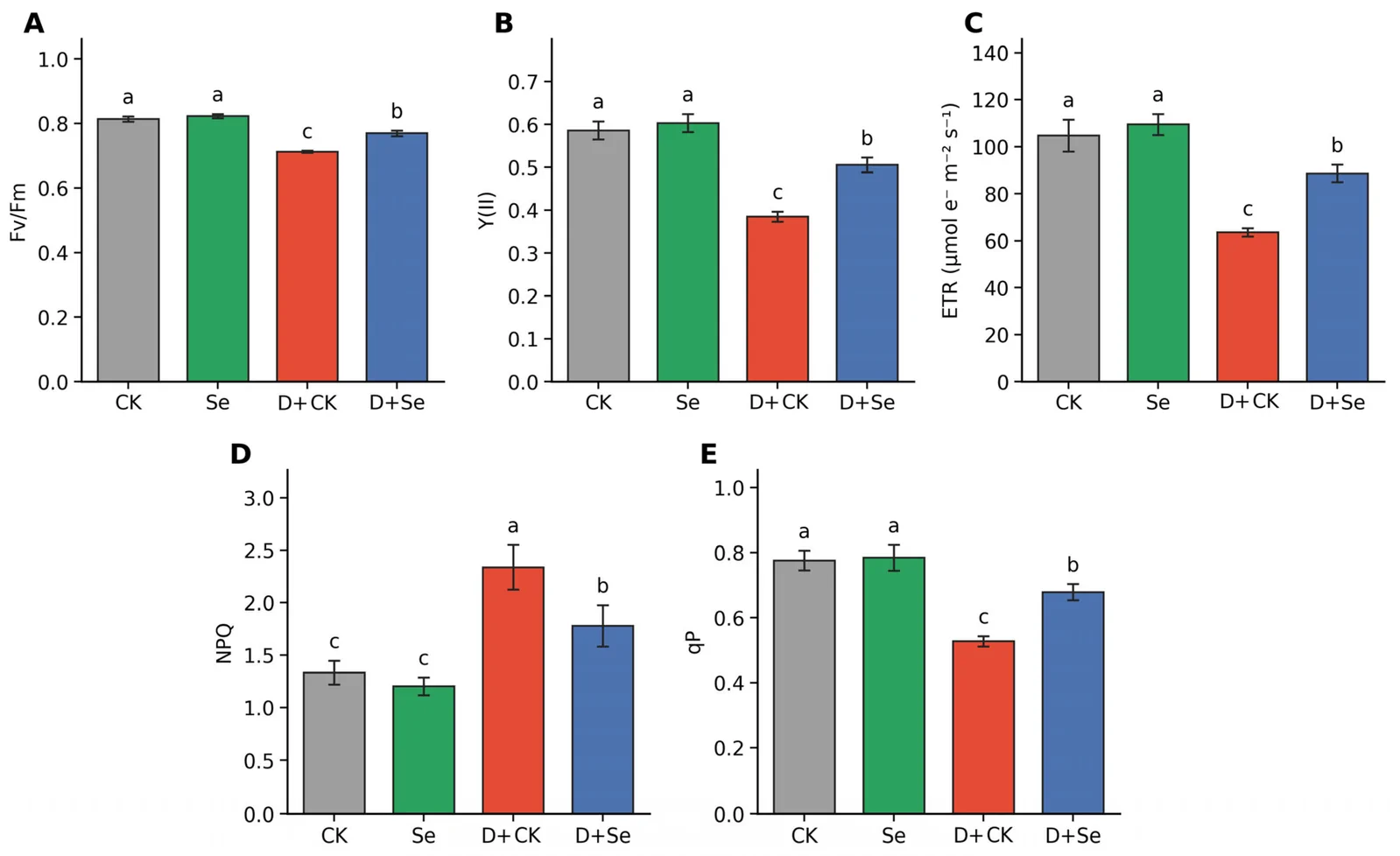
Effects of foliar sodium selenite application on PSII photochemical parameters of mulberry leaves under drought stress. Effects of different treatments on (**A**) Fv/Fm, (**B**) Y(II), (**C**) electron transport rate (ETR), (**D**) non-photochemical quenching (NPQ), and (**E**) photochemical quenching coefficient (qP). Values are means ± SD (n = 6). Different lowercase letters indicate significant differences among treatments according to Tukey’s HSD test at *p* < 0.05.

Chlorophyll fluorescence analysis showed that drought stress impaired PSII photochemical performance. Compared with CK, D+CK reduced Fv/Fm, Y(II), ETR, and qP by 12.5%, 34.4%, 39.3%, and 32.1%, respectively (Figure 3A–C,E). Meanwhile, NPQ increased by 75.3% under D+CK compared with CK (Figure 3D), indicating enhanced thermal energy dissipation under drought-induced excitation pressure. Two-way ANOVA showed significant effects of water regime, sodium selenite application, and their interaction on Fv/Fm, Y(II), ETR, NPQ, and qP (Table S1).

Foliar sodium selenite application partially mitigated drought-induced changes in PSII photochemical performance. Compared with D+CK, D+Se increased Fv/Fm, Y(II), ETR, and qP by 8.0%, 31.4%, 39.5%, and 28.6%, respectively. NPQ decreased by 23.9% in D+Se compared with D+CK. These results show that D+Se had higher PSII photochemical performance and lower NPQ than D+CK under drought stress.

### 3.4. Oxidative Damage and Antioxidant Enzymes

**Figure 4:**
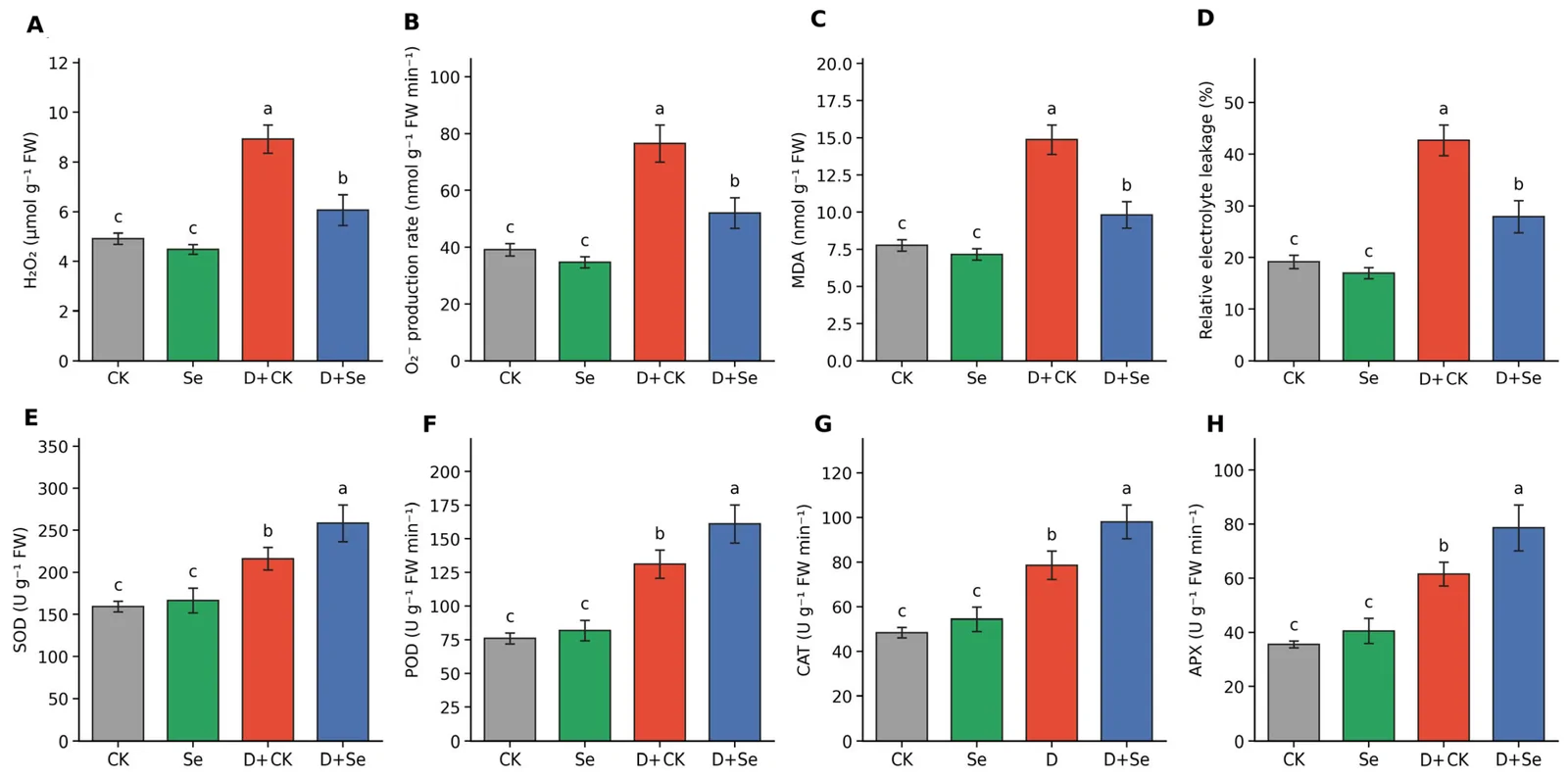
Effects of foliar sodium selenite application on oxidative-damage indicators and antioxidant enzyme activities of mulberry leaves under drought stress. Effects of different treatments on (**A**) H_2_O_2_ content, (**B**) O_2_^−^ production rate, (**C**) MDA content, (**D**) relative electrolyte leakage, (**E**) SOD activity, (**F**) POD activity, (**G**) CAT activity, and (**H**) APX activity. Values are means ± SD (n = 6). Different lowercase letters indicate significant differences among treatments according to Tukey’s HSD test at *p* < 0.05.

Drought stress caused marked oxidative damage in mulberry leaves. Compared with CK, D+CK increased H_2_O_2_ content, O_2_^−^ production rate, MDA content, and relative electrolyte leakage by 81.5%, 95.7%, 91.5%, and 122.9%, respectively (Figure 4A–D). These increases indicate that drought promoted ROS accumulation, membrane lipid peroxidation, and membrane permeability. Two-way ANOVA showed significant effects of water regime, sodium selenite application, and their interaction on oxidative-damage indicators (H_2_O_2_ content, O_2_^−^ production rate, MDA content, and relative electrolyte leakage) and antioxidant enzyme activities (SOD, POD, CAT, and APX) (Table S1).

Foliar sodium selenite application was associated with lower oxidative-damage indicators under drought stress. Compared with D+CK, D+Se decreased H_2_O_2_ content, O_2_^−^ production rate, MDA content, and relative electrolyte leakage by 32.0%, 32.0%, 34.0%, and 34.6%, respectively. Meanwhile, antioxidant enzyme activities were higher in D+Se than in D+CK. Compared with D+CK, D+Se increased SOD, POD, CAT, and APX activities by 19.5%, 22.9%, 24.8%, and 27.8%, respectively (Figure 4E–H).

### 3.5. Functional Compounds and Osmotic Adjustment

**Figure 5:**
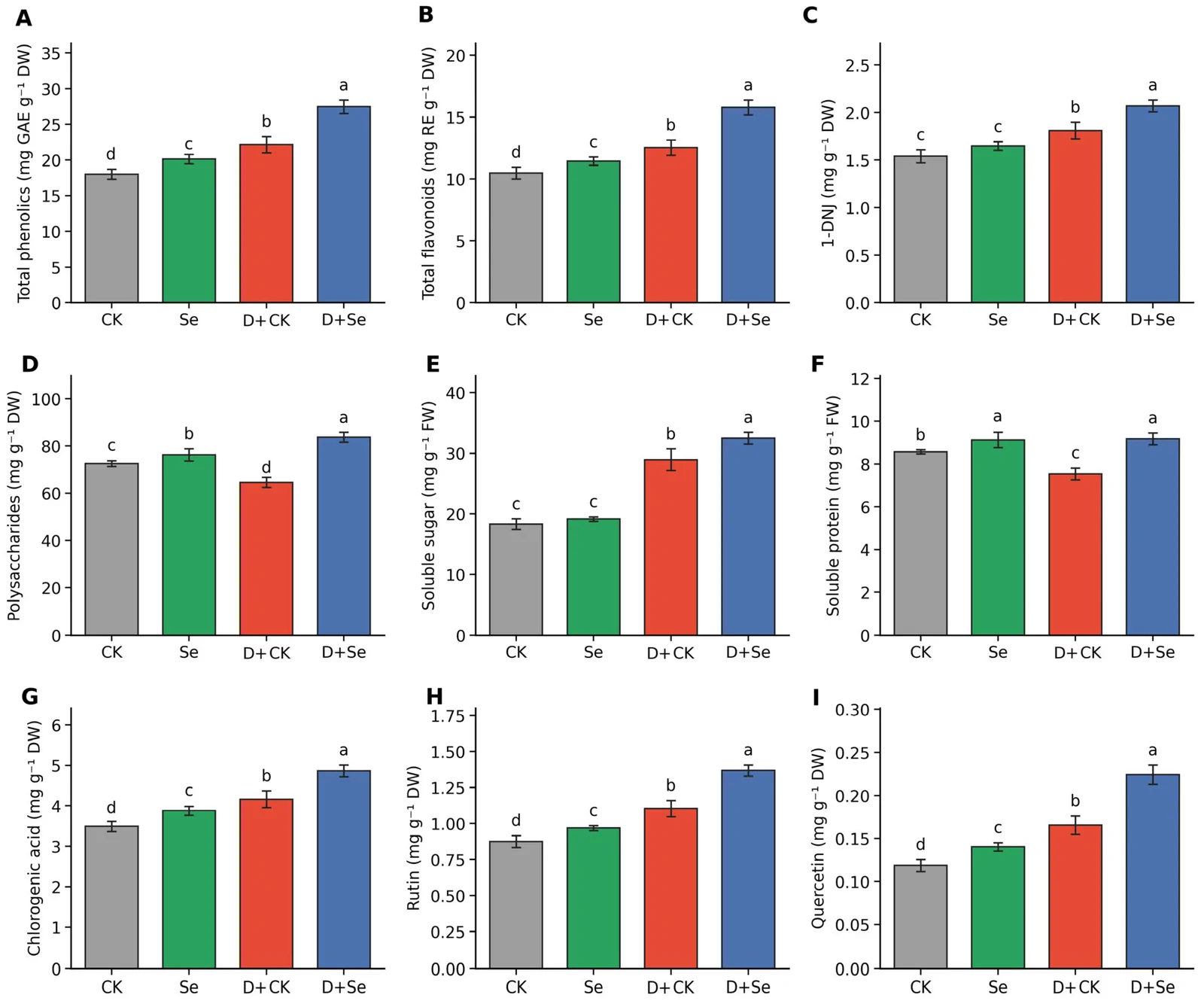
Effects of foliar sodium selenite application on functional compounds and osmotic-adjustment substances in mulberry leaves under drought stress. Effects of different treatments on (**A**) total phenolics, (**B**) total flavonoids, (**C**) 1-deoxynojirimycin (1-DNJ), (**D**) polysaccharides, (**E**) soluble sugar, (**F**) soluble protein, (**G**) chlorogenic acid, (**H**) rutin, and (**I**) quercetin. Values are means ± SD (n = 6). Different lowercase letters indicate significant differences among treatments according to Tukey’s HSD test at *p* < 0.05.

Drought stress and sodium selenite application significantly affected functional compounds in mulberry leaves. Compared with CK, D+CK increased total phenolics, total flavonoids, and 1-DNJ by 22.9%, 19.6%, and 17.6%, respectively (Figure 5A–C). Chlorogenic acid, rutin, and quercetin also increased by 19.2%, 26.0%, and 39.5%, respectively, under D+CK compared with CK (Figure 5G–I). These results suggest that drought stimulated the accumulation of several stress-responsive secondary metabolites. Two-way ANOVA showed significant effects of sodium selenite application on all measured secondary metabolites and osmotic/nutritional constituents (Table S1). Water regime significantly affected the measured secondary metabolites (total phenolics, total flavonoids, 1-DNJ, chlorogenic acid, rutin, and quercetin), as well as soluble sugar and soluble protein, but did not significantly affect polysaccharide content.

However, drought decreased polysaccharide and soluble protein contents by 11.0% and 12.0%, respectively, compared with CK (Figure 5D,F). Soluble sugar increased by 58.1% under D+CK, reflecting drought-induced osmotic adjustment (Figure 5E). Therefore, drought did not uniformly improve mulberry leaf quality; rather, it promoted some secondary metabolites while suppressing growth-related and nutritional components.

Foliar sodium selenite application increased several functional-quality indicators under drought stress. Compared with D+CK, D+Se increased total phenolics, total flavonoids, 1-DNJ, polysaccharides, soluble sugar, soluble protein, chlorogenic acid, rutin, and quercetin by 24.0%, 25.9%, 14.3%, 29.6%, 12.3%, 21.7%, 16.9%, 23.9%, and 35.4%, respectively.

### 3.6. Total Selenium Accumulation

**Figure 6:**
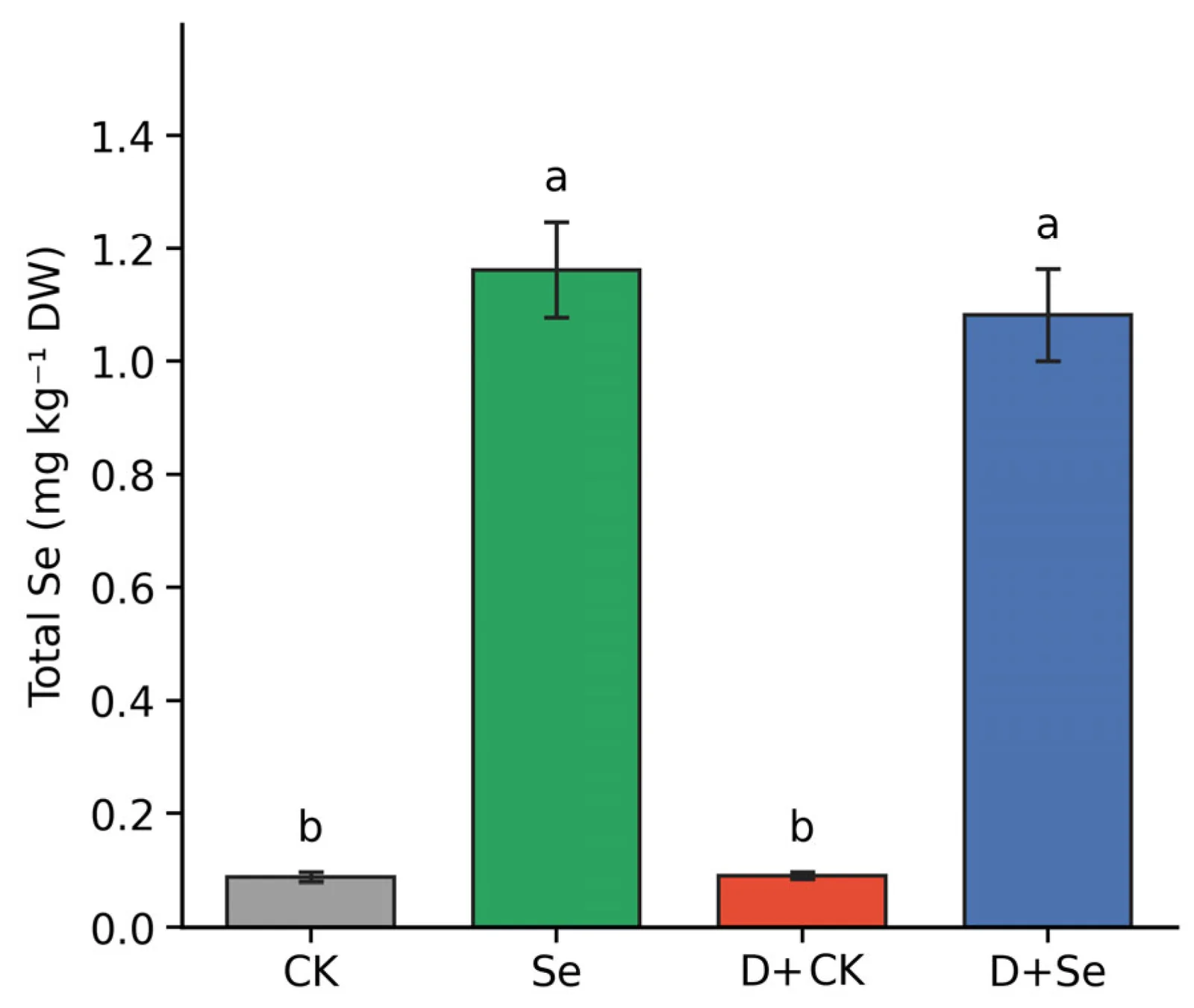
Effects of foliar sodium selenite application on total selenium content in mulberry leaves under drought stress. CK, normal water supply plus surfactant-containing control spray; Se, normal water supply with foliar sodium selenite application; D+CK, drought stress plus surfactant-containing control spray; D+Se, drought stress with foliar sodium selenite application. Values are means ± SD (n = 6). Different lowercase letters indicate significant differences among treatments according to Tukey’s HSD test at *p* < 0.05.

Foliar sodium selenite application substantially increased total selenium accumulation in mulberry leaves. Total Se content was low and similar in CK and D+CK, with values of 0.088 and 0.090 mg kg^−1^ DW, respectively (Figure 6). In contrast, Se and D+Se treatments increased total Se content to 1.162 and 1.082 mg kg^−1^ DW, respectively. Compared with CK, Se treatment increased total Se content by more than 12-fold, and compared with D+CK, D+Se increased total Se content by approximately 11-fold. Two-way ANOVA showed a strong selenium-application effect on total selenium content, whereas the water-regime effect and the water regime × selenium interaction were not significant (Table S1).

### 3.7. Antioxidant Capacity

**Figure 7:**
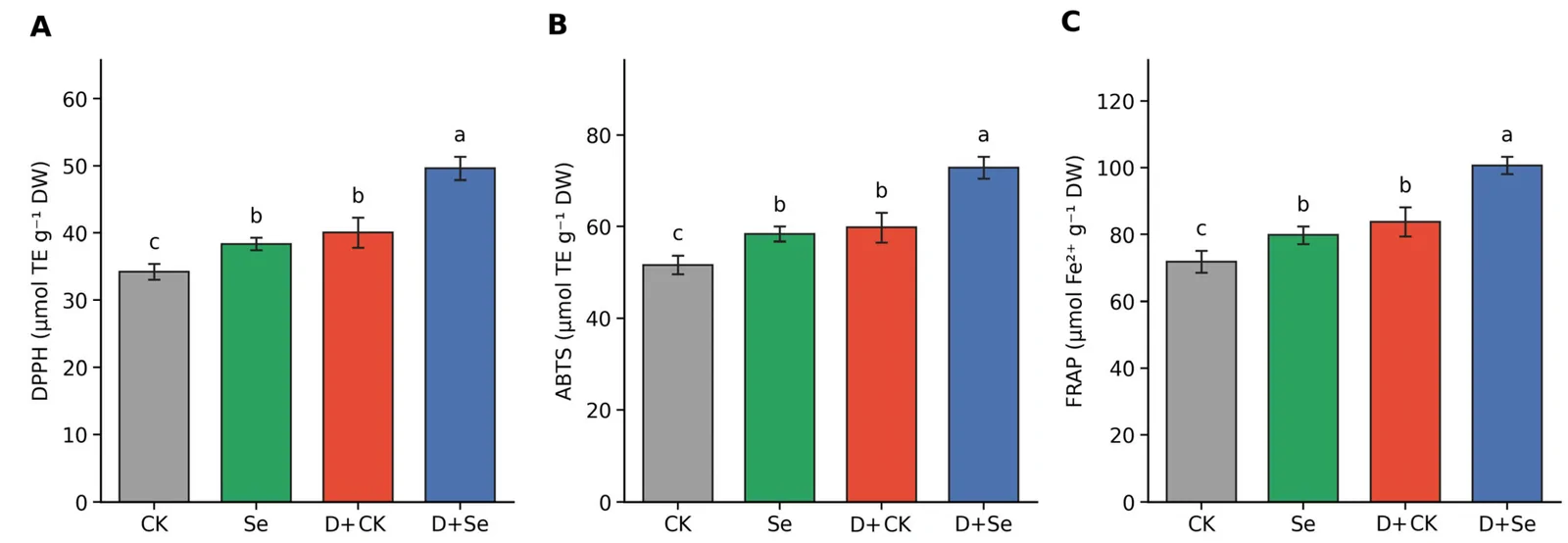
Effects of foliar sodium selenite application on the in vitro antioxidant capacity of mulberry leaves under drought stress. Effects of different treatments on (**A**) DPPH radical-scavenging capacity, (**B**) ABTS radical-scavenging capacity, and (**C**) FRAP reducing capacity. Values are means ± SD (n = 6). Different lowercase letters indicate significant differences among treatments according to Tukey’s HSD test at *p* < 0.05.

Drought stress increased the antioxidant capacity of mulberry leaf extracts. Compared with CK, D+CK increased DPPH, ABTS, and FRAP values by 17.0%, 15.8%, and 16.5%, respectively (Figure 7A–C). These increases were consistent with the higher levels of total phenolics, total flavonoids, chlorogenic acid, rutin, and quercetin under drought stress. Two-way ANOVA showed significant effects of water regime, sodium selenite application, and their interaction on all three in vitro antioxidant-capacity indices, namely DPPH, ABTS, and FRAP (Table S1).

Foliar sodium selenite application further increased the measured in vitro antioxidant-capacity indicators, particularly under drought stress. Compared with D+CK, D+Se increased DPPH, ABTS, and FRAP values by 24.0%, 21.8%, and 20.2%, respectively.

## 4. Discussion

### 4.1. Growth, Water Status, and Photosynthesis

Drought markedly restricted the growth of ‘Longsang No. 1’ mulberry seedlings and reduced leaf relative water content (Figure 1). The concurrent decreases in plant height, leaf area, and shoot biomass indicate that limited water availability constrained leaf expansion and biomass production. Similar reductions in leaf area and biomass have been reported in drought-stressed mulberry and may reduce transpirational demand while also limiting carbon assimilation [8]. The decrease in leaf relative water content further indicates that drought disrupted leaf water balance under the present experimental conditions.

Compared with D+CK, D+Se plants maintained higher leaf relative water content and greater biomass accumulation. These responses suggest that foliar sodium selenite application partially alleviated drought-related growth restriction and water deficit. The limited effects of sodium selenite application under normal water supply further indicate that the treatment primarily modified the response to drought rather than generally stimulating seedling growth. At low concentrations, selenium may influence plant stress responses through antioxidant defense, membrane stability, redox regulation, and photosynthetic processes [12,13].

Drought also impaired leaf pigment status and gas exchange (Figure 2). The decreases in Pn, g_s_, and Tr indicate restrictions on CO_2_ diffusion and transpiration. The concurrent increase in Ci, despite the decline in Pn, suggests that non-stomatal limitations also contributed to the inhibition of carbon assimilation. These limitations may have involved reduced biochemical CO_2_-assimilation capacity or impaired chloroplast and electron-transport processes. Foliar sodium selenite application partially maintained Pn, g_s_, and Tr and reduced the drought-induced increase in Ci. These concurrent responses suggest closer coordination between CO_2_ diffusion and assimilation in D+Se plants than in D+CK plants, although the present measurements do not establish a direct causal relationship among these variables.

Chlorophyll-fluorescence responses provided additional evidence of drought-related impairment of the photosynthetic apparatus (Figure 3). Lower Fv/Fm, Y(II), ETR, and qP indicate reductions in PSII efficiency, photochemical energy use, electron transport, and reaction-center openness. The increase in NPQ indicates greater dissipation of absorbed excitation energy as heat under drought-induced excitation pressure [44,45,46].

Compared with D+CK, D+Se plants showed higher Fv/Fm, Y(II), ETR, and qP and lower NPQ. These responses suggest that foliar sodium selenite application partially maintained PSII photochemical performance and allowed a greater proportion of absorbed light energy to remain available for photochemical use. The concurrent improvements in ETR, Y(II), and Pn were consistent with partial maintenance of photosynthetic electron transport and carbon assimilation. However, because these parameters were measured during the same experimental stage, their associations should not be interpreted as evidence of a confirmed temporal or causal pathway.

### 4.2. Oxidative Damage and Antioxidant Defense

Drought increased H_2_O_2_ content, O_2_^−^ production rate, MDA content, and relative electrolyte leakage in mulberry leaves (Figure 4). These responses indicate greater ROS accumulation, lipid peroxidation, and loss of membrane integrity. Water deficit can restrict photosynthetic carbon fixation and disrupt cellular electron transport, thereby increasing oxidative pressure in plant tissues [47].

Compared with D+CK, D+Se plants had lower H_2_O_2_, O_2_^−^, MDA, and relative electrolyte leakage but higher SOD, POD, CAT, and APX activities. SOD participates in the conversion of O_2_^−^ to H_2_O_2_, whereas CAT, POD, and APX contribute to H_2_O_2_ removal. The coordinated changes in these enzyme activities were, therefore, associated with lower oxidative-damage indicators in sodium-selenite-treated plants. Lower MDA content and electrolyte leakage also suggest partial maintenance of membrane stability, which may have been associated with the better leaf water status and photosynthetic performance observed in D+Se.

These findings do not establish that changes in any individual antioxidant enzyme directly caused the reductions in oxidative injury. Rather, they indicate that higher antioxidant enzyme activities and lower ROS-related damage occurred concurrently under the present treatment conditions.

Comparable responses have been reported in other crops. Foliar selenium application improved turgor maintenance, gas exchange, antioxidant-system activity, and yield-related performance in drought-stressed wheat [48]. In greenhouse tomato, foliar selenium application reduced MDA accumulation and partially maintained photosynthetic performance under drought stress [49]. Both sodium selenite and sodium selenate also improved growth, photosynthesis, osmotic adjustment, and antioxidant-system responses in drought-stressed tobacco [50]. These studies are broadly consistent with the present results, although direct comparisons are limited by differences in crop species, selenium form and concentration, application method, plant developmental stage, and drought treatment.

### 4.3. Functional Compounds, Selenium Enrichment, and In Vitro Antioxidant Capacity

Drought did not affect all quality-related constituents in the same direction (Figure 5). Total phenolics, total flavonoids, 1-DNJ, chlorogenic acid, rutin, and quercetin increased under drought, suggesting the accumulation of several stress-responsive secondary metabolites. Phenolic and flavonoid compounds may participate in antioxidant protection, and their involvement in the drought response of mulberry leaves has also been reported previously [51].

However, the accumulation of these compounds should not be interpreted as a uniform improvement in mulberry leaf quality. Drought simultaneously reduced seedling growth, leaf water status, photosynthetic performance, polysaccharide content, and soluble protein content and increased oxidative injury. The results, therefore, indicate a trade-off: drought promoted the accumulation of several stress-responsive secondary metabolites but impaired biomass production and overall physiological stability.

Foliar sodium selenite application modified this balance. Compared with D+CK, D+Se plants had higher contents of several secondary metabolites as well as higher polysaccharide, soluble sugar, and soluble protein contents. These findings suggest that sodium selenite application was associated with partial maintenance or accumulation of selected functional-quality indicators under drought. Mulberry leaves are known to contain phenolics, flavonoids, 1-DNJ, chlorogenic acid, rutin, and quercetin, which contribute to their phytochemical characteristics and chemical antioxidant properties [4,5,7,20].

Foliar sodium selenite application also markedly increased total selenium content under both water regimes, confirming selenium enrichment of the leaves (Figure 6). Nevertheless, total selenium content does not identify the selenium species present and cannot independently establish nutritional value, gastrointestinal bioaccessibility, or intake safety. Selenium speciation, processing stability, serving size, background dietary intake, and total selenium exposure would need to be considered before the food-oriented value or safety of selenium-enriched mulberry leaves could be evaluated.

DPPH, ABTS, and FRAP values were higher in D+Se than in D+CK (Figure 7). These changes were consistent with the higher concentrations of total phenolics, total flavonoids, chlorogenic acid, rutin, and quercetin. This correspondence suggests an association between phytochemical accumulation and the in vitro antioxidant capacity of the leaf extracts. However, the present data do not demonstrate that any individual compound directly caused the higher assay values.

DPPH and ABTS assess radical-scavenging capacity in different chemical reaction systems, whereas FRAP assesses ferric-ion-reducing power. These measurements, therefore, provide complementary information on in vitro antioxidant capacity. They should not be interpreted as direct evidence of antioxidant effects in humans, animals, or other in vivo systems.

### 4.4. Limitations and Implications

Under the present controlled conditions, foliar sodium selenite application was associated with partial maintenance of leaf water status, photosynthetic performance, PSII photochemical function, antioxidant enzyme activities, total selenium content, and selected functional-quality indicators under drought stress. These responses occurred concurrently but do not establish a confirmed temporal or causal pathway among the measured processes.

Several limitations should be considered. This study comprised a single 30 d controlled pot experiment and included only one mulberry cultivar, one sodium selenite concentration, and one drought intensity. Therefore, the observed responses should not be generalized across years, production environments, or mulberry genotypes. They should also not be interpreted as evidence that ‘Longsang No. 1’ has superior selenium-enrichment capacity relative to other cultivars.

Because no sodium selenite concentration gradient was included, the present results do not establish a dose–response relationship or identify an optimal application concentration. Lower or higher concentrations may produce different physiological, biochemical, and selenium-enrichment responses. The 10 μM treatment evaluated here should, therefore, not be regarded as an optimized or generally recommended application dose.

Root responses, selenium distribution among plant organs, individual selenium species, processing stability, gastrointestinal bioaccessibility, and intake safety were not assessed. Further experiments should evaluate multiple sodium selenite concentrations, drought intensities, mulberry cultivars, growing seasons, and production environments. Selenium speciation and field-scale performance should also be examined before practical recommendations are made.

Within these limitations, foliar sodium selenite application may be considered a potential experimental preharvest approach for simultaneously investigating drought-stress alleviation and selenium enrichment in mulberry leaves. Its practical value remains to be confirmed under production conditions.

## 5. Conclusions

Under the present controlled pot conditions, drought reduced growth, leaf water status, photosynthetic performance, and PSII function while increasing oxidative damage in ‘Longsang No. 1’ mulberry seedlings. Foliar application of 10 μM sodium selenite was associated with partial maintenance of these physiological processes, higher antioxidant enzyme activities, and lower oxidative-damage indicators under drought. The treatment also increased total selenium content and several functional-quality indicators, including selected phenolic compounds and in vitro antioxidant capacity. These findings support foliar sodium selenite application as a potential experimental preharvest treatment for partial drought-stress alleviation and selenium enrichment in mulberry leaves. However, dose optimization, selenium speciation, bioaccessibility, intake safety, cultivar-dependent responses, and field performance require further evaluation before practical recommendations can be made.

## Data Availability

The original contributions presented in the study are included in the article; further inquiries can be directed to the corresponding author.

## Supplementary Materials

The following supporting information can be downloaded at: https://www.mdpi.com/article/10.3390/biology15161368/s1, Table S1: Two-way ANOVA results for water regime, sodium selenite application, and their interaction; Table S2: One-way ANOVA results for the four treatment combinations.
