# Supplementary material for: Foliar Sodium Selenite Partially Alleviates Drought Injury and Improves Functional Quality in Mulberry Leaves
Source: Biology (Basel). 2026 Aug 11;15(16):1368. doi: 10.3390/biology15161368 (PMC13510285; doi:10.3390/biology15161368)
Supplement: Supplementary file 1 [file biology-15-01368-s001.zip › biology-4476536-supplementary.pdf]

Table S1. Two-way ANOVA results for the effects of water regime, selenium application, and their interaction on measured traits in 'Longsang No. 1' mulberry seedlings.

Water regime included normal water supply and drought stress; selenium application included foliar control spray and 10 µM sodium selenite. Each treatment combination contained six biological replicates (n = 6). Degrees of freedom are shown as numerator, denominator df. Exact p values and partial η^2 effect sizes are reported for significant and non-significant effects.

Category	Variable	Unit	Source	df	F	p	partial η^2
Growth	Plant height	cm	Water regime	1, 20	169.2734	3.2162×10^{-11}	0.8943
Growth	Plant height	cm	Selenium application	1, 20	35.4424	8.0257×10^{-6}	0.6393
Growth	Plant height	cm	Water regime × selenium application	1, 20	27.3481	4.0643×10^{-5}	0.5776
Growth	Leaf area	cm^2	Water regime	1, 20	159.5379	5.4694×10^{-11}	0.8886
Growth	Leaf area	cm^2	Selenium application	1, 20	29.8801	2.3762×10^{-5}	0.5990
Growth	Leaf area	cm^2	Water regime × selenium application	1, 20	29.2924	2.6845×10^{-5}	0.5943
Growth	Shoot fresh weight	g $plant^{-1}$	Water regime	1, 20	180.9351	1.7634×10^{-11}	0.9005
Growth	Shoot fresh weight	g $plant^{-1}$	Selenium application	1, 20	29.4971	2.5724×10^{-5}	0.5959
Growth	Shoot fresh weight	g $plant^{-1}$	Water regime × selenium application	1, 20	23.4827	9.8033×10^{-5}	0.5400
Growth	Shoot dry weight	g $plant^{-1}$	Water regime	1, 20	141.4981	1.5870×10^{-10}	0.8762
Growth	Shoot dry weight	g $plant^{-1}$	Selenium application	1, 20	21.6883	0.0002	0.5202
Growth	Shoot dry weight	g $plant^{-1}$	Water regime × selenium application	1, 20	14.7385	0.0010	0.4243
Water status	Leaf relative water content	%	Water regime	1, 20	380.7336	1.7291×10^{-14}	0.9501
Water status	Leaf relative water content	%	Selenium application	1, 20	79.6155	2.0742×10^{-8}	0.7992
Water status	Leaf relative water content	%	Water regime × selenium application	1, 20	52.1744	5.4405×10^{-7}	0.7229
Photosynthetic pigment	SPAD value	SPAD unit	Water regime	1, 20	343.6927	4.5719×10^{-14}	0.9450
Photosynthetic pigment	SPAD value	SPAD unit	Selenium application	1, 20	82.3393	1.5791×10^{-8}	0.8046

Category	Variable	Unit	Source	df	F	p	partial η²
Photosynthetic pigment	SPAD value	SPAD unit	Water regime × selenium application	1, 20	33.8473	1.0826×10^{-5}	0.6286
Photosynthetic pigment	Chlorophyll a	mg g^{-1} FW	Water regime	1, 20	235.3988	1.5855×10^{-12}	0.9217
Photosynthetic pigment	Chlorophyll a	mg g^{-1} FW	Selenium application	1, 20	56.6046	2.9697×10^{-7}	0.7389
Photosynthetic pigment	Chlorophyll a	mg g^{-1} FW	Water regime × selenium application	1, 20	26.9019	4.4814×10^{-5}	0.5736
Photosynthetic pigment	Chlorophyll b	mg g^{-1} FW	Water regime	1, 20	205.4253	5.5532×10^{-12}	0.9113
Photosynthetic pigment	Chlorophyll b	mg g^{-1} FW	Selenium application	1, 20	54.2715	4.0662×10^{-7}	0.7307
Photosynthetic pigment	Chlorophyll b	mg g^{-1} FW	Water regime × selenium application	1, 20	13.0358	0.0017	0.3946
Photosynthetic pigment	Carotenoids	mg g^{-1} FW	Water regime	1, 20	107.3706	1.7342×10^{-9}	0.8430
Photosynthetic pigment	Carotenoids	mg g^{-1} FW	Selenium application	1, 20	39.2923	4.0373×10^{-6}	0.6627
Photosynthetic pigment	Carotenoids	mg g^{-1} FW	Water regime × selenium application	1, 20	9.9957	0.0049	0.3332
Gas exchange	Net photosynthetic rate (Pn)	μmol CO_2 m^{-2} s^{-1}	Water regime	1, 20	295.4798	1.9032×10^{-13}	0.9366
Gas exchange	Net photosynthetic rate (Pn)	μmol CO_2 m^{-2} s^{-1}	Selenium application	1, 20	66.6998	8.4590×10^{-8}	0.7693
Gas exchange	Net photosynthetic rate (Pn)	μmol CO_2 m^{-2} s^{-1}	Water regime × selenium application	1, 20	31.3338	1.7685×10^{-5}	0.6104
Gas exchange	Stomatal conductance (Gs)	mmol H_2O m^{-2} s^{-1}	Water regime	1, 20	591.4433	2.5078×10^{-16}	0.9673
Gas exchange	Stomatal conductance (Gs)	mmol H_2O m^{-2} s^{-1}	Selenium application	1, 20	96.0275	4.4437×10^{-9}	0.8276
Gas exchange	Stomatal conductance (Gs)	mmol H_2O m^{-2} s^{-1}	Water regime × selenium application	1, 20	50.4961	6.9116×10^{-7}	0.7163
Gas exchange	Intercellular CO_2 concentration (Ci)	μmol mol^{-1}	Water regime	1, 20	74.5108	3.5315×10^{-8}	0.7884
Gas exchange	Intercellular CO_2 concentration (Ci)	μmol mol^{-1}	Selenium application	1, 20	27.7494	3.7254×10^{-5}	0.5811

Category	Variable	Unit	Source	df	F	p	partial η^2
Gas exchange	Intercellular CO_2 concentration (Ci)	μmol mol^{-1}	Water regime × selenium application	1, 20	7.6044	0.0121	0.2755
Gas exchange	Transpiration rate (Tr)	mmol H_2O m^{-2} s^{-1}	Water regime	1, 20	313.0159	1.1062×10^{-13}	0.9399
Gas exchange	Transpiration rate (Tr)	mmol H_2O m^{-2} s^{-1}	Selenium application	1, 20	54.3302	4.0336×10^{-7}	0.7309
Gas exchange	Transpiration rate (Tr)	mmol H_2O m^{-2} s^{-1}	Water regime × selenium application	1, 20	40.0479	3.5472×10^{-6}	0.6669
Gas exchange	Instantaneous water-use efficiency (WUE)	μmol CO_2 mmol^{-1} H_2O	Water regime	1, 20	3.3040	0.0841	0.1418
Gas exchange	Instantaneous water-use efficiency (WUE)	μmol CO_2 mmol^{-1} H_2O	Selenium application	1, 20	8.9346	0.0073	0.3088
Gas exchange	Instantaneous water-use efficiency (WUE)	μmol CO_2 mmol^{-1} H_2O	Water regime × selenium application	1, 20	0.0016	0.9682	0.0001
Chlorophyll fluorescence	Maximum quantum efficiency of PSII (Fv/Fm)	dimensionless	Water regime	1, 20	750.7813	2.4670×10^{-17}	0.9741
Chlorophyll fluorescence	Maximum quantum efficiency of PSII (Fv/Fm)	dimensionless	Selenium application	1, 20	137.5035	2.0420×10^{-10}	0.8730
Chlorophyll fluorescence	Maximum quantum efficiency of PSII (Fv/Fm)	dimensionless	Water regime × selenium application	1, 20	71.0035	5.1785×10^{-8}	0.7802
Chlorophyll fluorescence	Effective quantum yield of PSII [Y(II)]	dimensionless	Water regime	1, 20	399.1901	1.1011×10^{-14}	0.9523
Chlorophyll fluorescence	Effective quantum yield of PSII [Y(II)]	dimensionless	Selenium application	1, 20	84.8453	1.2366×10^{-8}	0.8092
Chlorophyll fluorescence	Effective quantum yield of PSII [Y(II)]	dimensionless	Water regime × selenium application	1, 20	47.8793	1.0158×10^{-6}	0.7054
Chlorophyll fluorescence	Electron transport rate (ETR)	μmol e^{-} m^{-2} s^{-1}	Water regime	1, 20	277.5576	3.4220×10^{-13}	0.9328
Chlorophyll fluorescence	Electron transport rate (ETR)	μmol e^{-} m^{-2} s^{-1}	Selenium application	1, 20	64.0896	1.1531×10^{-7}	0.7622
Chlorophyll fluorescence	Electron transport rate (ETR)	μmol e^{-} m^{-2} s^{-1}	Water regime × selenium application	1, 20	29.8537	2.3892×10^{-5}	0.5988
Chlorophyll fluorescence	Non-photochemical quenching (NPQ)	dimensionless	Water regime	1, 20	144.5860	1.3116×10^{-10}	0.8785

Category	Variable	Unit	Source	df	F	p	partial η^2
Chlorophyll fluorescence	Non-photochemical quenching (NPQ)	dimensionless	Selenium application	1, 20	27.4996	3.9325×10^{-5}	0.5789
Chlorophyll fluorescence	Non-photochemical quenching (NPQ)	dimensionless	Water regime × selenium application	1, 20	10.6486	0.0039	0.3474
Chlorophyll fluorescence	Photochemical quenching coefficient (qP)	dimensionless	Water regime	1, 20	224.7464	2.4317×10^{-12}	0.9183
Chlorophyll fluorescence	Photochemical quenching coefficient (qP)	dimensionless	Selenium application	1, 20	45.2218	1.5259×10^{-6}	0.6934
Chlorophyll fluorescence	Photochemical quenching coefficient (qP)	dimensionless	Water regime × selenium application	1, 20	36.2471	6.9243×10^{-6}	0.6444
Oxidative damage	Hydrogen peroxide (H_2O_2)	μmol g^{-1} FW	Water regime	1, 20	235.3980	1.5855×10^{-12}	0.9217
Oxidative damage	Hydrogen peroxide (H_2O_2)	μmol g^{-1} FW	Selenium application	1, 20	81.2439	1.7606×10^{-8}	0.8025
Oxidative damage	Hydrogen peroxide (H_2O_2)	μmol g^{-1} FW	Water regime × selenium application	1, 20	44.0304	1.8415×10^{-6}	0.6876
Oxidative damage	Superoxide anion production rate ($O_2{\bullet}^-$)	nmol g^{-1} FW min^{-1}	Water regime	1, 20	225.0573	2.4009×10^{-12}	0.9184
Oxidative damage	Superoxide anion production rate ($O_2{\bullet}^-$)	nmol g^{-1} FW min^{-1}	Selenium application	1, 20	62.5933	1.3834×10^{-7}	0.7578
Oxidative damage	Superoxide anion production rate ($O_2{\bullet}^-$)	nmol g^{-1} FW min^{-1}	Water regime × selenium application	1, 20	30.0773	2.2817×10^{-5}	0.6006
Oxidative damage	Malondialdehyde (MDA)	nmol g^{-1} FW	Water regime	1, 20	277.3537	3.4457×10^{-13}	0.9327
Oxidative damage	Malondialdehyde (MDA)	nmol g^{-1} FW	Selenium application	1, 20	93.4775	5.5612×10^{-9}	0.8238
Oxidative damage	Malondialdehyde (MDA)	nmol g^{-1} FW	Water regime × selenium application	1, 20	57.5786	2.6121×10^{-7}	0.7422
Membrane stability	Relative electrolyte leakage	%	Water regime	1, 20	335.7872	5.6989×10^{-14}	0.9438
Membrane stability	Relative electrolyte leakage	%	Selenium application	1, 20	81.2877	1.7529×10^{-8}	0.8025
Membrane stability	Relative electrolyte leakage	%	Water regime × selenium application	1, 20	45.0376	1.5706×10^{-6}	0.6925

Category	Variable	Unit	Source	df	F	p	partial η²
Antioxidant enzyme	Superoxide dismutase (SOD)	U g^{-1} FW	Water regime	1, 20	143.5475	1.3979×10^{-10}	0.8777
Antioxidant enzyme	Superoxide dismutase (SOD)	U g^{-1} FW	Selenium application	1, 20	15.8997	0.0007	0.4429
Antioxidant enzyme	Superoxide dismutase (SOD)	U g^{-1} FW	Water regime × selenium application	1, 20	7.8284	0.0111	0.2813
Antioxidant enzyme	Peroxidase (POD)	U g^{-1} FW min^{-1}	Water regime	1, 20	279.9930	3.1533×10^{-13}	0.9333
Antioxidant enzyme	Peroxidase (POD)	U g^{-1} FW min^{-1}	Selenium application	1, 20	20.0008	0.0002	0.5000
Antioxidant enzyme	Peroxidase (POD)	U g^{-1} FW min^{-1}	Water regime × selenium application	1, 20	9.0176	0.0070	0.3108
Antioxidant enzyme	Catalase (CAT)	U g^{-1} FW min^{-1}	Water regime	1, 20	243.1993	1.1722×10^{-12}	0.9240
Antioxidant enzyme	Catalase (CAT)	U g^{-1} FW min^{-1}	Selenium application	1, 20	28.8318	2.9570×10^{-5}	0.5904
Antioxidant enzyme	Catalase (CAT)	U g^{-1} FW min^{-1}	Water regime × selenium application	1, 20	8.1635	0.0097	0.2899
Antioxidant enzyme	Ascorbate peroxidase (APX)	U g^{-1} FW min^{-1}	Water regime	1, 20	216.1621	3.4802×10^{-12}	0.9153
Antioxidant enzyme	Ascorbate peroxidase (APX)	U g^{-1} FW min^{-1}	Selenium application	1, 20	25.7458	5.7989×10^{-5}	0.5628
Antioxidant enzyme	Ascorbate peroxidase (APX)	U g^{-1} FW min^{-1}	Water regime × selenium application	1, 20	7.7370	0.0115	0.2789
Functional quality	Total phenolics	mg GAE g^{-1} DW	Water regime	1, 20	246.5566	1.0322×10^{-12}	0.9250
Functional quality	Total phenolics	mg GAE g^{-1} DW	Selenium application	1, 20	104.1888	2.2381×10^{-9}	0.8390
Functional quality	Total phenolics	mg GAE g^{-1} DW	Water regime × selenium application	1, 20	19.0510	0.0003	0.4878
Functional quality	Total flavonoids	mg RE g^{-1} DW	Water regime	1, 20	225.7178	2.3369×10^{-12}	0.9186
Functional quality	Total flavonoids	mg RE g^{-1} DW	Selenium application	1, 20	98.1331	3.7061×10^{-9}	0.8307
Functional quality	Total flavonoids	mg RE g^{-1} DW	Water regime × selenium application	1, 20	28.7365	3.0171×10^{-5}	0.5896
Functional quality	1-Deoxynojirimycin (1-DNJ)	mg g^{-1} DW	Water regime	1, 20	155.5253	6.8654×10^{-11}	0.8861
Functional quality	1-Deoxynojirimycin (1-DNJ)	mg g^{-1} DW	Selenium application	1, 20	43.8237	1.9032×10^{-6}	0.6866

Category	Variable	Unit	Source	df	F	p	partial η^2
Functional quality	1-Deoxynojirimycin (1-DNJ)	mg g^{-1} DW	Water regime × selenium application	1, 20	7.2104	0.0142	0.2650
Functional quality	Polysaccharides	mg g^{-1} DW	Water regime	1, 20	0.0958	0.7601	0.0048
Functional quality	Polysaccharides	mg g^{-1} DW	Selenium application	1, 20	186.9264	1.3126×10^{-11}	0.9033
Functional quality	Polysaccharides	mg g^{-1} DW	Water regime × selenium application	1, 20	85.7083	1.1383×10^{-8}	0.8108
Osmotic adjustment	Soluble sugar	mg g^{-1} FW	Water regime	1, 20	687.1214	5.8481×10^{-17}	0.9717
Osmotic adjustment	Soluble sugar	mg g^{-1} FW	Selenium application	1, 20	23.0269	0.0001	0.5352
Osmotic adjustment	Soluble sugar	mg g^{-1} FW	Water regime × selenium application	1, 20	8.7277	0.0078	0.3038
Osmotic adjustment	Soluble protein	mg g^{-1} FW	Water regime	1, 20	19.6915	0.0003	0.4961
Osmotic adjustment	Soluble protein	mg g^{-1} FW	Selenium application	1, 20	97.8538	3.7957×10^{-9}	0.8303
Osmotic adjustment	Soluble protein	mg g^{-1} FW	Water regime × selenium application	1, 20	23.7606	9.1772×10^{-5}	0.5430
Antioxidant capacity	DPPH antioxidant capacity	μmol TE g^{-1} DW	Water regime	1, 20	172.3777	2.7310×10^{-11}	0.8960
Antioxidant capacity	DPPH antioxidant capacity	μmol TE g^{-1} DW	Selenium application	1, 20	111.1836	1.2881×10^{-9}	0.8475
Antioxidant capacity	DPPH antioxidant capacity	μmol TE g^{-1} DW	Water regime × selenium application	1, 20	17.6171	0.0004	0.4683
Antioxidant capacity	ABTS antioxidant capacity	μmol TE g^{-1} DW	Water regime	1, 20	132.8626	2.7594×10^{-10}	0.8692
Antioxidant capacity	ABTS antioxidant capacity	μmol TE g^{-1} DW	Selenium application	1, 20	101.6587	2.7546×10^{-9}	0.8356
Antioxidant capacity	ABTS antioxidant capacity	μmol TE g^{-1} DW	Water regime × selenium application	1, 20	10.3639	0.0043	0.3413
Antioxidant capacity	FRAP antioxidant capacity	μmol Fe^{2+} equivalents g^{-1} DW	Water regime	1, 20	145.9267	1.2088×10^{-10}	0.8795
Antioxidant capacity	FRAP antioxidant capacity	μmol Fe^{2+} equivalents g^{-1} DW	Selenium application	1, 20	84.1878	1.3177×10^{-8}	0.8080
Antioxidant capacity	FRAP antioxidant capacity	μmol Fe^{2+} equivalents g^{-1} DW	Water regime × selenium application	1, 20	11.0838	0.0033	0.3566

Category	Variable	Unit	Source	df	F	p	partial η^2
Selenium enrichment	Total selenium	mg kg^{-1} DW	Water regime	1, 20	2.6178	0.1213	0.1157
Selenium enrichment	Total selenium	mg kg^{-1} DW	Selenium application	1, 20	1836.0055	3.7380×10^{-21}	0.9892
Selenium enrichment	Total selenium	mg kg^{-1} DW	Water regime × selenium application	1, 20	2.9168	0.1031	0.1273
Functional quality	Chlorogenic acid	mg g^{-1} DW	Water regime	1, 20	178.6386	1.9793×10^{-11}	0.8993
Functional quality	Chlorogenic acid	mg g^{-1} DW	Selenium application	1, 20	77.0215	2.7086×10^{-8}	0.7939
Functional quality	Chlorogenic acid	mg g^{-1} DW	Water regime × selenium application	1, 20	6.5008	0.0191	0.2453
Functional quality	Rutin	mg g^{-1} DW	Water regime	1, 20	352.1848	3.6274×10^{-14}	0.9463
Functional quality	Rutin	mg g^{-1} DW	Selenium application	1, 20	114.3129	1.0156×10^{-9}	0.8511
Functional quality	Rutin	mg g^{-1} DW	Water regime × selenium application	1, 20	26.2048	5.2305×10^{-5}	0.5671
Functional quality	Quercetin	mg g^{-1} DW	Water regime	1, 20	321.9443	8.4843×10^{-14}	0.9415
Functional quality	Quercetin	mg g^{-1} DW	Selenium application	1, 20	121.6866	5.9274×10^{-10}	0.8588
Functional quality	Quercetin	mg g^{-1} DW	Water regime × selenium application	1, 20	25.8140	5.7104×10^{-5}	0.5635

Table S2. One-way ANOVA and Tukey's HSD comparisons among the four treatment combinations for measured traits in 'Longsang No. 1' mulberry seedlings.

Values are means ± SD (n = 6). CK, normal water supply plus control spray; Se, normal water supply plus 10 μM sodium selenite; D, drought stress plus control spray; D+Se, drought stress plus 10 μM sodium selenite. Different lowercase letters within a row indicate significant differences according to Tukey's HSD test at $p < 0.05$. The one-way analysis was used to support the lowercase letters presented in the figures and did not replace the factorial analysis in Table S1.

Category	Variable	Unit	CK	Se	D	D+Se	df	F	p	partial η²
Growth	Plant height	cm	72.03 ± 3.33 a	72.70 ± 1.80 a	55.23 ± 1.51 c	65.53 ± 1.95 b	3, 20	77.3546	3.5249×10^{-11}	0.9207
Growth	Leaf area	cm^2	128.33 ± 9.25 a	128.47 ± 4.60 a	83.80 ± 3.41 c	110.65 ± 5.28 b	3, 20	72.9035	6.0617×10^{-11}	0.9162
Growth	Shoot fresh weight	g $plant^{-1}$	58.13 ± 5.05 a	58.89 ± 2.23 a	34.53 ± 1.78 c	47.79 ± 2.51 b	3, 20	77.9717	3.2772×10^{-11}	0.9212
Growth	Shoot dry weight	g $plant^{-1}$	12.40 ± 1.00 a	12.60 ± 0.36 a	8.59 ± 0.23 c	10.65 ± 0.47 b	3, 20	59.3083	3.9137×10^{-10}	0.8990
Water status	Leaf relative water content	%	87.27 ± 2.62 a	88.57 ± 1.27 a	66.82 ± 1.62 c	79.17 ± 1.71 b	3, 20	170.8412	2.0304×10^{-14}	0.9624
Photosynthetic pigment	SPAD value	SPAD unit	40.72 ± 1.00 a	41.97 ± 1.25 a	31.37 ± 0.59 c	37.08 ± 0.79 b	3, 20	153.2931	5.7386×10^{-14}	0.9583
Photosynthetic pigment	Chlorophyll a	mg g^{-1} FW	1.76 ± 0.08 a	1.82 ± 0.08 a	1.25 ± 0.02 c	1.57 ± 0.03 b	3, 20	106.3018	1.8440×10^{-12}	0.9410
Photosynthetic pigment	Chlorophyll b	mg g^{-1} FW	0.583 ± 0.029 a	0.617 ± 0.029 a	0.419 ± 0.009 c	0.519 ± 0.015 b	3, 20	90.9109	7.9411×10^{-12}	0.9317
Photosynthetic pigment	Carotenoids	mg g^{-1} FW	0.326 ± 0.015 a	0.340 ± 0.011 a	0.264 ± 0.010 c	0.308 ± 0.008 b	3, 20	52.2195	1.2122×10^{-9}	0.8868
Gas exchange	Net photosynthetic rate (Pn)	μmol CO_2 m^{-2} s^{-1}	14.57 ± 0.89 a	15.36 ± 1.01 a	7.58 ± 0.21 c	11.80 ± 0.62 b	3, 20	131.1711	2.5343×10^{-13}	0.9516
Gas exchange	Stomatal conductance (Gs)	mmol H_2O m^{-2} s^{-1}	244.58 ± 15.66 a	256.38 ± 12.02 a	106.90 ± 5.25 c	180.97 ± 6.58 b	3, 20	245.9889	5.9806×10^{-16}	0.9736
Gas exchange	Intercellular CO_2 concentration (Ci)	μmol mol^{-1}	277.33 ± 9.51 bc	266.85 ± 5.37 c	324.90 ± 10.51 a	291.38 ± 13.74 b	3, 20	36.6215	2.5754×10^{-8}	0.8460
Gas exchange	Transpiration rate (Tr)	mmol H_2O m^{-2} s^{-1}	4.10 ± 0.24 a	4.18 ± 0.29 a	2.18 ± 0.07 c	3.28 ± 0.08 b	3, 20	135.7980	1.8230×10^{-13}	0.9532
Gas exchange	Instantaneous water-use efficiency (WUE)	μmol CO_2 $mmol^{-1}$ H_2O	3.55 ± 0.06 ab	3.68 ± 0.10 a	3.48 ± 0.05 b	3.60 ± 0.16 ab	3, 20	4.0801	0.0206	0.3797
Chlorophyll fluorescence	Maximum quantum efficiency of PSII (Fv/Fm)	dimensionless	0.814 ± 0.008 a	0.823 ± 0.006 a	0.712 ± 0.003 c	0.769 ± 0.009 b	3, 20	319.7627	4.6275×10^{-17}	0.9796

Category	Variable	Unit	CK	Se	D	D+Se	df	F	p	partial η²
Chlorophyll fluorescence	Effective quantum yield of PSII [Y(II)]	dimensionless	0.586 ± 0.021 a	0.603 ± 0.021 a	0.385 ± 0.012 c	0.505 ± 0.017 b	3, 20	177.3049	1.4208×10^{-14}	0.9638
Chlorophyll fluorescence	Electron transport rate (ETR)	μmol e^{-} m^{-2} s^{-1}	104.75 ± 6.81 a	109.48 ± 4.47 a	63.55 ± 1.72 c	88.63 ± 3.74 b	3, 20	123.8337	4.3738×10^{-13}	0.9489
Chlorophyll fluorescence	Non-photochemical quenching (NPQ)	dimensionless	1.33 ± 0.11 c	1.20 ± 0.08 c	2.34 ± 0.21 a	1.78 ± 0.20 b	3, 20	60.9114	3.0821×10^{-10}	0.9013
Chlorophyll fluorescence	Photochemical quenching coefficient (qP)	dimensionless	0.776 ± 0.030 a	0.784 ± 0.039 a	0.527 ± 0.016 c	0.678 ± 0.025 b	3, 20	102.0718	2.6979×10^{-12}	0.9387
Oxidative damage	Hydrogen peroxide (H_2O_2)	μmol g^{-1} FW	4.92 ± 0.23 c	4.48 ± 0.20 c	8.92 ± 0.57 a	6.07 ± 0.62 b	3, 20	120.2241	5.7860×10^{-13}	0.9475
Oxidative damage	Superoxide anion production rate ($O_2^{\bullet-}$)	nmol g^{-1} FW min^{-1}	39.12 ± 2.15 c	34.68 ± 1.95 c	76.53 ± 6.52 a	52.07 ± 5.40 b	3, 20	105.9093	1.9091×10^{-12}	0.9408
Oxidative damage	Malondialdehyde (MDA)	nmol g^{-1} FW	7.77 ± 0.38 c	7.16 ± 0.38 c	14.88 ± 0.99 a	9.82 ± 0.89 b	3, 20	142.8033	1.1292×10^{-13}	0.9554
Membrane stability	Relative electrolyte leakage	%	19.15 ± 1.29 c	16.98 ± 1.06 c	42.68 ± 2.97 a	27.90 ± 3.10 b	3, 20	154.0375	5.4788×10^{-14}	0.9585
Antioxidant enzyme	Superoxide dismutase (SOD)	U g^{-1} FW	159.25 ± 6.34 c	166.63 ± 14.85 c	216.25 ± 13.41 b	258.37 ± 22.00 a	3, 20	55.7585	6.7846×10^{-10}	0.8932
Antioxidant enzyme	Peroxidase (POD)	U g^{-1} FW min^{-1}	76.00 ± 4.12 c	81.90 ± 7.65 c	131.13 ± 10.52 b	161.15 ± 14.17 a	3, 20	103.0038	2.4778×10^{-12}	0.9392
Antioxidant enzyme	Catalase (CAT)	U g^{-1} FW min^{-1}	48.42 ± 2.34 c	54.37 ± 5.47 c	78.58 ± 6.39 b	98.07 ± 7.64 a	3, 20	93.3982	6.1790×10^{-12}	0.9334
Antioxidant enzyme	Ascorbate peroxidase (APX)	U g^{-1} FW min^{-1}	35.55 ± 1.24 c	40.55 ± 4.64 c	61.55 ± 4.39 b	78.68 ± 8.47 a	3, 20	83.2149	1.8007×10^{-11}	0.9258
Functional quality	Total phenolics	mg GAE g^{-1} DW	18.02 ± 0.70 d	20.14 ± 0.66 c	22.15 ± 1.16 b	27.45 ± 0.96 a	3, 20	123.2655	4.5684×10^{-13}	0.9487
Functional quality	Total flavonoids	mg RE g^{-1} DW	10.48 ± 0.48 d	11.45 ± 0.34 c	12.54 ± 0.61 b	15.78 ± 0.61 a	3, 20	117.5291	7.1675×10^{-13}	0.9463
Functional quality	1-Deoxynojirimycin (1-DNJ)	mg g^{-1} DW	1.54 ± 0.07 c	1.65 ± 0.04 c	1.81 ± 0.09 b	2.07 ± 0.06 a	3, 20	68.8531	1.0198×10^{-10}	0.9117
Functional quality	Polysaccharides	mg g^{-1} DW	72.60 ± 1.20 c	76.28 ± 2.53 b	64.62 ± 2.13 d	83.75 ± 2.08 a	3, 20	90.9102	7.9417×10^{-12}	0.9317
Osmotic adjustment	Soluble sugar	mg g^{-1} FW	18.30 ± 0.87 c	19.15 ± 0.37 c	28.94 ± 1.78 b	32.48 ± 0.98 a	3, 20	239.6254	7.7160×10^{-16}	0.9729

Category	Variable	Unit	CK	Se	D	D+Se	df	F	p	partial η²
Osmotic adjustment	Soluble protein	mg g^{-1} FW	8.57 ± 0.09 b	9.12 ± 0.36 a	7.54 ± 0.28 c	9.17 ± 0.28 a	3, 20	47.1020	2.9925×10^{-9}	0.8760
Antioxidant capacity	DPPH antioxidant capacity	μmol TE g^{-1} DW	34.22 ± 1.18 c	38.35 ± 0.91 b	40.03 ± 2.23 b	49.63 ± 1.73 a	3, 20	100.3928	3.1506×10^{-12}	0.9377
Antioxidant capacity	ABTS antioxidant capacity	μmol TE g^{-1} DW	51.65 ± 2.05 c	58.38 ± 1.62 b	59.80 ± 3.23 b	72.85 ± 2.41 a	3, 20	81.6284	2.1503×10^{-11}	0.9245
Antioxidant capacity	FRAP antioxidant capacity	μmol Fe^{2+} equivalents g^{-1} DW	71.90 ± 3.30 c	79.82 ± 2.61 b	83.75 ± 4.40 b	100.68 ± 2.63 a	3, 20	80.3994	2.4725×10^{-11}	0.9234
Selenium enrichment	Total selenium	mg kg^{-1} DW	0.088 ± 0.008 b	1.16 ± 0.08 a	0.090 ± 0.006 b	1.08 ± 0.08 a	3, 20	613.8467	7.5451×10^{-20}	0.9893
Functional quality	Chlorogenic acid	mg g^{-1} DW	3.49 ± 0.13 d	3.88 ± 0.11 c	4.16 ± 0.21 b	4.87 ± 0.15 a	3, 20	87.3870	1.1457×10^{-11}	0.9291
Functional quality	Rutin	mg g^{-1} DW	0.876 ± 0.042 d	0.969 ± 0.017 c	1.10 ± 0.06 b	1.37 ± 0.04 a	3, 20	164.2342	2.9650×10^{-14}	0.9610
Functional quality	Quercetin	mg g^{-1} DW	0.119 ± 0.007 d	0.140 ± 0.005 c	0.166 ± 0.011 b	0.224 ± 0.011 a	3, 20	156.4816	4.7126×10^{-14}	0.9591
